# Precision targeting of FDX1-mediated cuproptosis by a ROS-responsive hydrogel for myocardial ischemia-reperfusion injury treatment

**DOI:** 10.7150/thno.120455

**Published:** 2026-01-01

**Authors:** Jiayi Hu, Xiaoyi Bao, Meihua Ting, Yecheng Tao, Ran Li, Guosheng Fu, Fuyu Qiu, Jing Zhao, Wenbin Zhang

**Affiliations:** Zhejiang Key Laboratory of Cardiovascular Intervention and Precision Medicine, Engineering Research Center for Cardiovascular Innovative Devices of Zhejiang Province, Department of Cardiology, Sir Run Run Shaw Hospital, Zhejiang University School of Medicine, Hangzhou, 310016, China.

**Keywords:** myocardial ischemia-reperfusion injury, cuproptosis, oxidative stress, ferredoxin 1, ROS-responsive & scavenging

## Abstract

**Rationale:** Myocardial ischemia-reperfusion injury (MIRI) poses a critical clinical challenge due to intertwined oxidative stress and cuproptosis-driven cell death. Current therapies inadequately address dual-pathology mechanisms (ROS overproduction and copper dysregulation), while conventional drug delivery lacks spatiotemporal precision.

**Methods:** A ROS-responsive hydrogel (OD@G4CAsi-FDX1) was engineered via dynamic Schiff base crosslinking between oxidized dextran (OD) and polyamidoamine dendrimers (PAMAM G4). The hydrogel co-encapsulates antioxidant caffeic acid (CA) and FDX1-targeted siRNA (si-FDX1). *In vitro* biocompatibility, ROS scavenging, and mitochondrial protection were assessed in primary cardiomyocytes. *In vivo* efficacy was evaluated in a murine MIRI model following intramyocardial hydrogel injection. Cardiac function, infarct size, and molecular markers were analyzed.

**Results:** *In vitro*, it reduced ROS, preserved mitochondrial membrane potential, and suppressed pro-inflammatory cytokines. *In vivo*, it reduced infarct size, suppressed cuproptosis markers, and improved cardiac function. Mechanistically, si-FDX1 blocked DLAT oligomerization, while CA neutralized ROS, synergistically restoring redox homeostasis. This efficacy was enabled by sustained ROS-triggered release.

**Conclusions:** OD@G4CAsi-FDX1 hydrogel dual-targets ROS and cuproptosis via a single injectable platform, overcoming limitations of conventional mono-mechanistic therapies. It demonstrates significant cardioprotection and clinical potential for MIRI management.

## Introduction

Myocardial ischemia-reperfusion injury (MIRI) remains a major clinical challenge in acute myocardial infarction (MI) management, significantly worsening patient outcomes. The pathophysiology of MIRI is complex, involving mitochondrial dysfunction, oxidative stress, and inflammatory cascades 1. Mitochondria, the cellular powerhouses responsible for energy production, are essential for maintaining the functional stability of cardiomyocytes. Mitochondrial dysfunction not only amplifies oxidative damage via excessive reactive oxygen species (ROS) production 2 but also activates multiple cell death pathways, including apoptosis and necrosis 3. In turn, these pathways can further exacerbate mitochondrial damage, creating a self-reinforcing cycle that ultimately leads to cardiac functional impairment 4.

Cuproptosis is a novel form of regulated cell death characterized by copper dependency and associated with mitochondrial metabolism. Copper ions (Cu ions), essential for cellular respiration and energy metabolism, are tightly regulated to maintain normal cellular function. 5. When dysregulation of Cu homeostasis occurs, Cu ions bind to lipoylated enzymes in tricarboxylic acid (TCA), which leads to their aggregation, proteotoxic stress, and ultimately cell death 6. Cu ions exist in both monovalent (Cu^+^) and divalent (Cu^2+^) forms in biological systems. Relevant to their toxicity, Cu^2+^ tend to form coordination bonds with intracellular molecules, such as DNA and lipids 7, while Cu^+^ can form stable complexes with thiol groups (such as cysteine residues) within cells, disrupting protein structure and function 8. While cuproptosis has been implicated in ischemic damage models 9, its role in MIRI remains to be fully elucidated.

The iron-sulfur cluster protein Ferredoxin 1 (FDX1), a key reductase in mitochondrial electron transfer, has emerged as a critical mediator of Cu^+^/Cu^2+^ homeostasis and cuproptosis. As an essential component of cellular redox reactions, FDX1 facilitates the reduction of Cu^2+^ to the more cytotoxic Cu^+^. This intracellularly accumulation of Cu^+^ triggers the aggregation of lipoylated proteins and destabilizing Fe-S cluster proteins 10. FDX1-mediated Cu^+^ production disrupts mitochondrial function and exacerbates oxidative stress, thereby accelerating cell death 11. Recent studies have highlighted its pivotal role in the pathogenesis of both cerebral IRI 9 and cardiovascular diseases 12. However, further in-depth research on the mechanism of FDX1 in MIRI and its therapeutic targeting is urgently needed.

Polyphenolic compounds, such as CA and its derivatives, exhibit potent antioxidant properties, effectively scavenging ROS, mitigating oxidative stress, and inhibiting inflammatory cascades 13. Studies have demonstrated that CA reduces myocardial fibrosis and enhances cardiac function following MI 14. Building on these antioxidant strategies, ROS-responsive hydrogels can be tailored to address the pathological overproduction of ROS in the injured myocardium after IR, achieving precise targeting and sustained drug release in the damaged area through myocardial injection 15. Stable hydrogel networks with ROS responsiveness can be constructed by utilizing Schiff base reactions between oxidized polysaccharides and dendritic macromolecules 16. However, the clinical translation of MIRI therapies is hindered by the pathological complexity of the condition and the inherent limitations of current delivery paradigms. Specifically, the efficacy of conventional therapeutics is severely compromised by their short half-lives, low bioavailability, and lack of target specificity. Systemic administration further contends with rapid clearance by the reticuloendothelial system (e.g., liver and spleen sequestration) and the relentless washout effect of continuous cardiac blood flow, preventing adequate drug accumulation at the infarct site. Moreover, prevailing single-mode treatment strategies are not only insufficient to address the dynamic fluctuations of ROS and the interplay of novel cell death pathways. Even advanced antioxidative nanodrugs often fall short due to an inability to comprehensively cover all relevant oxidative stress pathways, resulting in limited protective effects.

This critical limitation underscores the pressing need for innovative combinatorial strategies capable of concurrently targeting multiple key pathological pathways in MIRI. To address this challenge, we developed a unique polyamidoamine dendrimers-based hydrogel system (OD@G4CAsi-FDX1) that pioneeringly integrates antioxidant therapy with gene silencing against cuproptosis. This platform uses fourth-generation polyamidoamine (PAMAM G4) dendrimers to co-deliver CA, an antioxidant 17, and FDX1-targeting small interfering RNA (si-FDX1), which suppresses aberrant copper metabolism by downregulating FDX1 expression 18. The hydrogel is formed via dynamic Schiff base crosslinking between G4CAsi-FDX1 and oxidized dextran (OD), creating a stable network that enables controlled and sustained release of both the drug (CA) and gene (si-FDX1) components in response to elevated ROS levels at the lesion site. The released si-FDX1 downregulates FDX1 expression, thereby inhibiting the Cu^2+^ to Cu^+^ conversion and preserving mitochondrial integrity. Concurrently, CA rapidly scavenges burst ROS, creating a favorable microenvironment for si-FDX1 function. This combination is anticipated to amplify the protective effects against myocardial injury in a mouse model of MIRI. Additionally, the hydrogel's long-term retention ensures sustained drug/gene delivery, further augmenting its therapeutic potential. This study presents a ROS-responsive hydrogel for myocardial repair that simultaneously targets ROS burst and cuproptosis to disrupt the injury cycle, highlighting promising clinical prospects.

## Materials and Methods

### Oxidation degree of OD

The oxidation degree of OD was determined by a hydroxylamine hydrochloride titration method. The aldehyde groups in OD react with NH_2_OH·HCl, releasing equimolar H+ ions that were titrated with standardized NaOH using methyl orange as an indicator. A blank titration was performed for correction.

### Encapsulation efficiency (EE) determination of CA

The EE% of CA was determined using ultraviolet-visible spectroscopy (UV-Vis, Shimadzu UV-2600). The solvent was removed under reduced pressure. Cold distilled water was then added to dissolve the G4CA. The mixture was centrifuged to remove free CA (which is insoluble in water). The precipitate was collected and dissolved in methanol, and the amount of unencapsulated CA was determined by UV-Vis spectroscopy based on its characteristic absorption at 232 nm. The EE was then calculated as follows: EE% = (Amount of encapsulated CA / Total feeding amount of CA) × 100%.

### Preparation and characterization of OD@G4CAsi-FDX1 hydrogel

CA (Aladdin) was dissolved in Methanol and gradually added to a PAMAM G4 (Stargray, Xi'an XingGray Biotechnology Co., Ltd.) aqueous solution (mass ratio 1:1) under stirring at room temperature for 24 h in the dark, forming a drug-loaded dendritic macromolecular complex (G4CA). Subsequently, si-FDX1 was dissolved in DEPC-treated water and mixed with the complex solution at a mass ratio of (0.2-5):1 at room temperature for 30 min to achieve electrostatic adsorption (G4CAsi-FDX1). Finally, OD, prepared by reacting dextran (HEOWNS, Xi'an Heowns Biotech Co., Ltd.) with sodium periodate (NaIO_4_, SCR/Shanghai Chemical Reagents Co.) at a 1:1 molar ratio for 6 h, was rapidly mixed with the composite solution (G4:OD = 1:1 mass ratio, 5% w/v) at room temperature, resulting in a ROS-responsive hydrogel (OD@G4CAsi-FDX1). This hydrogel network is formed through dynamic covalent cross-linking between aminated PAMAM G4 dendrimers and aldehyde-modified OD via Schiff base chemistry. Under mild physiological conditions, primary amine groups (-NH_2_) on the dendrimers react with aldehyde groups (-CHO) on OD, forming imine bonds (-N=CH-) and reversible linkages. Along with physical entanglements of dextran chains, this reaction constructs a three-dimensional covalent network that provides structural integrity and dynamic properties.

### Mechanical and physical characterization of the hydrogel

The swelling ratio was determined gravimetrically after equilibrating hydrogels in deionized water at 37 °C for 24 h. Porosity was measured using a compression-weight method based on the mass difference before and after removing interstitial water. Rheological properties, including viscoelasticity, collapse and self-healing behavior, were characterized by oscillatory frequency sweeps (0.1-10 rad/s), strain amplitude sweeps (0.1-1000%), and step-strain tests using a rotational rheometer. The compressive modulus was obtained from uniaxial compression tests on cylindrical samples at a rate of 10 mm/min. Ex vivo adhesion strength was evaluated by measuring the shear resistance of hydrogel-tissue interfaces after 24 h equilibration. Detailed protocols are provided in the Supporting Information.

### Cell culture and treatment

Neonatal rat cardiomyocytes (NRCMs), extracted from 1-day-old rats, were grown in Dulbecco's Modified Eagle Medium (DMEM, D0819, Sigma-Aldrich, MO, USA) including 10% (v/v) fetal bovine serum (FBS, F8192, Sigma-Aldrich), 1% (v/v) penicillin-streptomycin solution (15070-063, Gibco, CA, USA) at 37 °C in the incubator (MCO-5AC, Panasonic, Kyoto, Japan) with 5% CO_2_ and 95% air. To stimulate MIRI, NRCMs were cultured in hypoxia incubator with 94% N_2_, 5% CO_2_ and 1% O_2_ for 4 h, and then were cultured in normal incubator with 5% CO_2_ and 95% air.

### Animal model of MIRI

MIRI was induced in 8-week-old male C57BL/6J mice anesthetized with 3% isoflurane (1 L/min) and maintained on a thermostatic plate at 37℃. Following precordial sterilization, a 2-cm thoracic incision was made at the point of maximal cardiac impulse to access the thoracic cavity through the fourth intercostal space. The left anterior descending coronary artery (LAD) was occluded with a 7-0 polypropylene slipknot for 45 minutes, after which reperfusion was initiated by releasing the knot. All animals were closely monitored during postoperative recovery. All animal experiments were approved by the Tab of Animal Experiment Ethical Inspection of the Sir Run Run Shaw Hospital, Zhejiang University School of Medicine (SRRSH2025-0037).

### *In vivo* degradation of ICG-labeled hydrogel

Indocyanine Green (ICG, MCE) was first dissolved in saline, and the ICG was then mixed with the hydrogel prior to injection. The labeled hydrogel was injected into mice via the epicardial myocardium under open-chest surgery. The degradation process of the hydrogel was monitored using the *in vivo* imaging system (IVIS, Lumina LT, Perkin Elmer). Imaging time points were typically set at 0, 0.5, 1, 2, 3, 4, 5, 6, 24, 36, 48, and 72 h post-injection. The near-infrared fluorescence channel of the IVIS was used to observe the degradation of the hydrogel *in vivo*, allowing for analysis of the hydrogel's local stability and degradation rate.

### *In vivo* release and distribution of Cy5-labeled G4CAsi-FDX1 NPs

Cy5-NHS (Cy5 N-Hydroxysuccinimide ester, Aladdin) was first conjugated to the PAMAM G4 dendrimer. The labeled NPs was then injected into mice via the epicardial myocardium under open-chest surgery. Post-injection, real-time imaging was performed using the IVIS (Lumina LT, Perkin Elmer). Imaging time points were set according to experimental requirements, typically at 0, 0.5, 1, 2, 3, 4, 5, 6, 24, 36, 48, and 72 h after drug injection. The distribution of the labeled drug was detected using the Cy5 fluorescence channel of the IVIS, enabling analysis of the drug's release and distribution *in vivo*.

## Results and Discussion

### Copper dysregulation and FDX1-driven mitochondrial damage in MIRI

**Figure 1:**
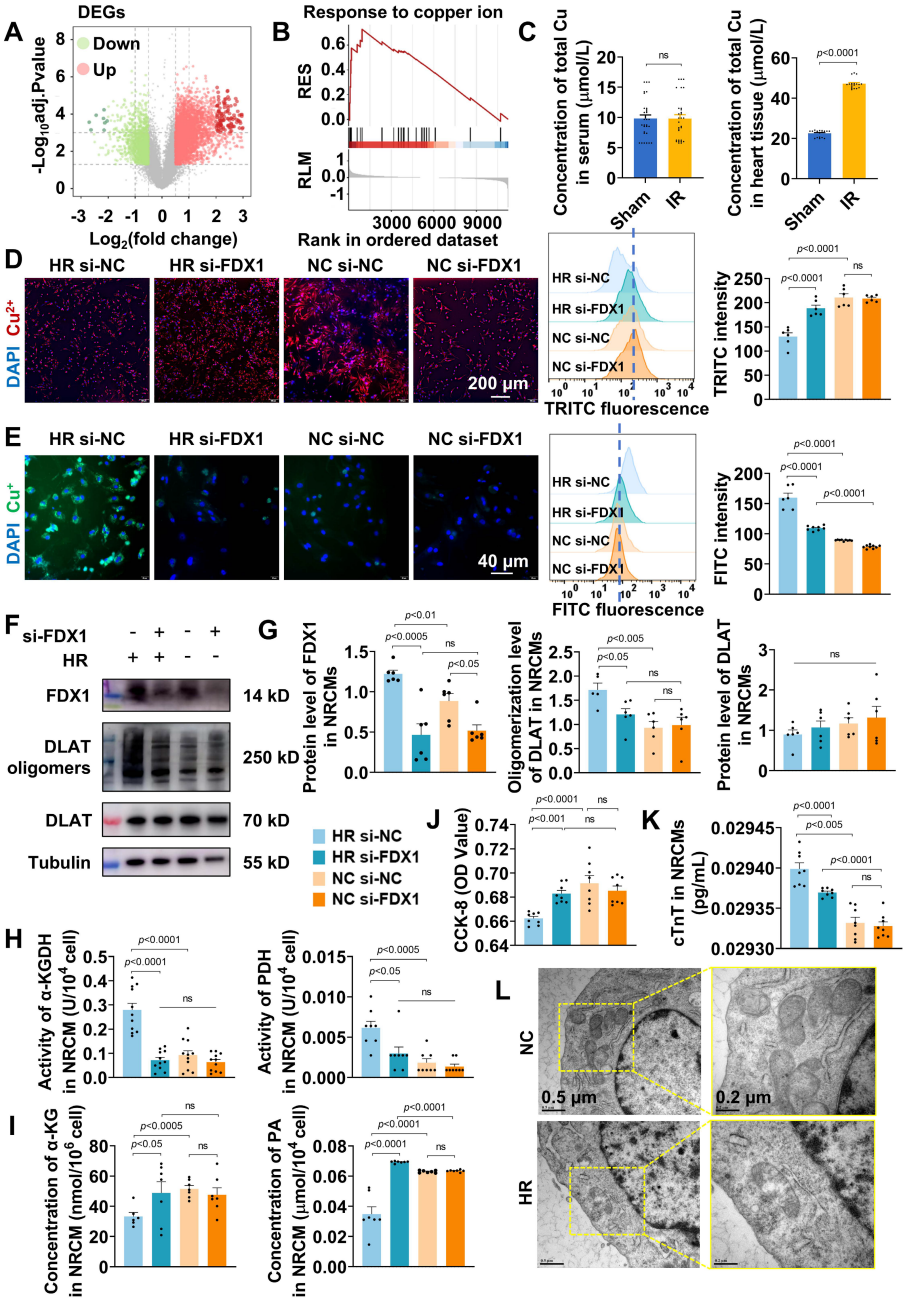
Copper dysregulation and FDX1-driven mitochondrial damage in MIRI. A) DEGs in the IRI model compared to the sham operation group were shown by volcano plot (Red dots: upregulated genes, green dots: downregulated genes). B) GSEA demonstrating enrichment of the “response to copper ions” gene set in the ranked list of DEGs from (A). C) Total Cu concentration in serum and in the heart tissue from the sham operation and IRI groups (n = 30). D) Representative Cu^2+^ (red) staining of NRCMs from different groups (scale bar: 200 µm). Flow cytometric profile and quantitative results (n = 6). E) Representative Cu^+^ (green) staining of NRCMs from different groups (scale bar: 40 µm). Flow cytometric profile and quantitative results (n ≥ 6). F, G) Protein levels of FDX1 (14 kD), DLAT oligomers (250 kD) and DLAT (70 kD) in NRCMs after different treatments. The left panels are WB bands and the right panels are quantitative data (n = 6). H) Activity of α-KGDH and PDH in NRCMs after different treatments. I) Concentrations of α-KG and PA in NRCMs after different treatments. J) Cell viability was measured with the CCK-8 assay from different groups. K) Concentration of cTNT in NRCMs was detected from different groups. L) TEM analysis of mitochondrial morphology in HR-treated NRCMs (scale bar: 0.5 μm and 0.2 μm). For quantitative data (H-K), data are mean ± s.e.m. (n ≥ 6). Statistical significance is indicated by *p* < 0.05. P values were determined using one-way ANOVA with Tukey's post hoc analysis.

To identify potential biomarkers involved in MIRI, we analyzed the GEO dataset (GSE193997) and applied stringent screening criteria, including |LogFC| > 0.5 and p-value < 0.05, to identify MIRI-related DEGs. A volcano plot was generated to visualize the DEGs following the comparison between the MIRI and sham groups (**Figure 1**A). In the samples collected at 6 h post-MIRI, a total of 7,694 DEGs were identified that met the aforementioned criteria. GSEA was conducted to further explore the biological characteristics of these DEGs at the 6-h time point. Notably, GSEA revealed a significant enrichment in the "response to copper ion" pathway (Figure 1B), highlighting the potential involvement of copper ions in MIRI. To investigate copper ion dynamics, we examined the total copper increased by 110% post-MIRI (Figure 1C), with Cu^2+^ levels rising 67% in hypoxia-reoxygenation (HR) treated cardiomyocytes (Figure S1). Further analysis using specific probes for Cu^+^ and Cu^2+^ ions revealed that, after reperfusion, Cu^2+^ levels decreased 38% (Figure 1D), whereas Cu^+^ levels rose 1.8-fold (Figure 1E), both of which can be disrupted by si-FDX1. This alteration may be due to the reduction of Cu^2+^ to Cu^+^ by the electron donor FDX1.

Next, we assessed FDX1 expression in MIRI mouse heart tissues. Boxplot analysis showed significant upregulation of Fdx1 in the MIRI group compared to the sham group (Figure S2). Additionally, in primary rat cardiomyocytes, FDX1 expression was also elevated after HR (Figure 1F, 1G, S3). The observed elevation in FDX1 levels was closely associated with increased oligomerization of dihydrolipoamide S-acetyltransferase (DLAT), a key subunit that regulates alpha-ketoglutarate dehydrogenase (α-KGDH) 19 and pyruvate dehydrogenase (PDH) activity 20. While DLAT mRNA levels decreased in HR (Figure S3), its protein oligomers significantly increased (Figure 1F, 1G). FDX1 knockdown suppressed DLAT oligomerization, indicating FDX1's regulatory role in DLAT stability and TCA cycle activity. We further observed an enhanced activity of α-KGDH and PDH (Figure 1H), and a decrease in the levels of alpha-ketoglutarate (α-KG) and pyruvate (PA) after HR (Figure 1I). The enhanced enzyme activity may be compensating for a disrupted metabolic pathway, further contributing to the generation of ROS and exacerbating mitochondrial dysfunction in MIRI.

Following HR insult, cellular viability exhibited a significant decline (Figure 1J, S4), accompanied by a notable elevation in the cardiac injury marker cardiac troponin T (cTnT) (Figure 1K). Finally, electron microscopy analysis revealed a reduction in the mitochondrial inner membrane and the formation of large vacuoles in the MIRI group, which was partially alleviated following FDX1 knockdown (Figure 1L), establishing FDX1 as a central mediator linking copper metabolism and mitochondrial dysfunction in HR.

### Synthesis and characterization of ROS-responsive hydrogel

**Figure 2:**
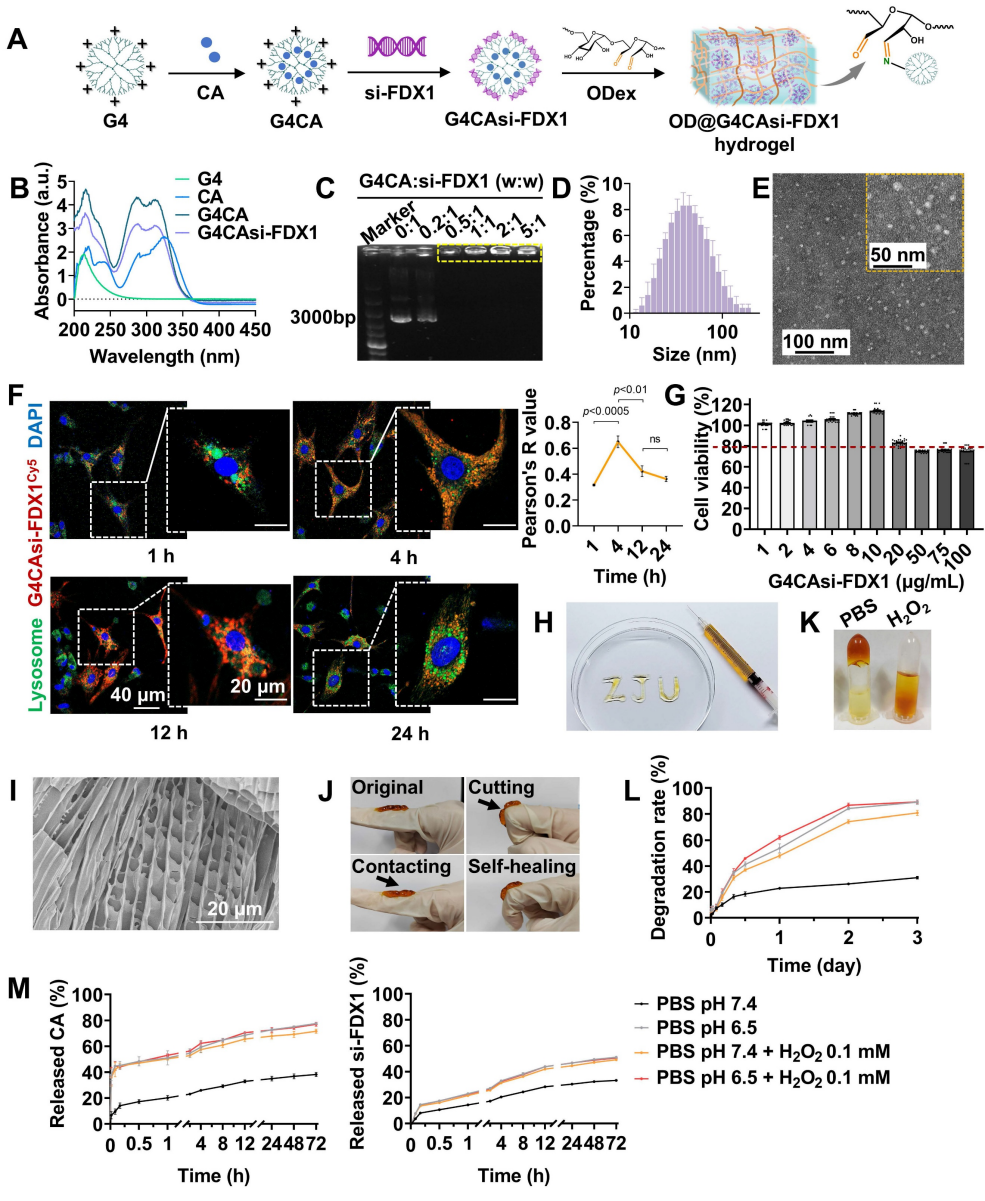
Synthesis and characterization of ROS-responsive hydrogel. A) Schematic design of OD@G4CAsi-FDX1 hydrogel. Integration of ROS-cleavable Schiff base bonds (enabling ROS-triggered degradation) and G4CAsi-FDX1 NPs (providing dual drug/gene delivery). B) UV/Vis spectra validating component integration. C) Agarose gel electrophoresis of si-FDX1 complexation efficiency (G4CA:si-FDX1 w:w ratios, from 0.2:1 to 5:1). D) Hydrodynamic size distribution of G4CAsi-FDX1 NPs. E) TEM images of G4CAsi-FDX1 NPs (scale bar: 100 nm and 50 nm). F) Confocal images showing intracellular trafficking of G4CAsi-FDX1 NPs in NRCMs after 1, 4, 12, 24 h of incubation (scale bar: 40 μm and 20 μm) and co-localization quantification via Pearson's correlation coefficient (n = 3). Late endosomes and lysosomes were stained with LysoTracker (green), while nuclei were stained with DAPI (blue). G) Cell viability of NRCMs treated by G4CAsi-FDX1 NPs with different concentration. H) Injectability of the hydrogel. I) Representative SEM image of the freeze-dried hydrogel (scale bar: 20 μm). J) Self-healing property of the hydrogel. K) Macroscopic observation of the ROS responsiveness of the hydrogel. L) Kinetic degradation curve of OD@G4CAsi-FDX1 hydrogel in PBS (pH 7.4), PBS (pH 6.5), PBS (pH 7.4) + 0.1 mM H_2_O_2_, and PBS (pH 6.5) + 0.1 mM H_2_O_2_ (n = 3). M) Release kinetics of CA and si-FDX1 from OD@G4CAsi-FDX1 hydrogel in PBS (pH 7.4), PBS (pH 6.5), PBS (pH 7.4) + 0.1 mM H_2_O_2_, and PBS (pH 6.5) + 0.1 mM H_2_O_2_ (n = 3).

Based on the findings that FDX1 upregulation and Cu^+^/Cu^2+^ imbalance exacerbate mitochondrial dysfunction in MIRI, we engineered a ROS-responsive hydrogel system to simultaneously scavenge ROS and inhibit FDX1-mediated cuproptosis (**Figure 2**A). The system utilized G4 dendrimers as carriers for co-encapsulating CA and electrostatically adsorbing si-FDX1, forming nanoparticles (NPs) designated G4CAsi-FDX1. These NPs were subsequently cross-linked with OD (oxidation degree: 54.64%) via dynamic Schiff base bonds, with the reaction between the dendrimers' primary amine groups (-NH_2_) and OD's aldehyde groups (-CHO) forming imine bonds (-N=CH-) to yield the ROS-responsive hydrogel OD@G4CAsi-FDX1.

The G4CAsi-FDX1 NPs were prepared by encapsulating CA into G4 dendrimers followed by electrostatic absorption of si-FDX1. The EE of CA was ~85% (Figure S5). UV-Vis spectroscopy identified characteristic absorption peaks for both G4 and CA, indicating successful drug loading (Figure 2B). Agarose gel electrophoresis demonstrated complete si-FDX1 complexation at a 0.5:1 mass ratio (G4CA:si-FDX1) (Figure 2C). Dynamic light scattering (DLS) analysis of NPs (G4CA:si-FDX1 = 2:1) revealed a hydrodynamic diameter of 58.70 ± 2.45 nm (Figure 2D) and a zeta potential of + 38.8 mV (Figure S6). Transmission electron microscopy (TEM) imaging further confirmed the formation of monodisperse spherical NPs (Figure 2E).

Cellular uptake assays indicated that when the ratio of G4CA to si-FDX1 was 2:1, G4CAsi-FDX1 NPs at a concentration of 1 μg/mL resulted in over 50% of cells exhibiting internalized NPs, confirming effective drug delivery (Figure S7). Confocal microscopy using Cy5-labeled NPs demonstrated their intracellular trafficking: within 1 h, NPs were internalized into cells and localized in endosomes; by 4 h, they had reached lysosomes; by 12 h, they remained in lysosomes and slowly degraded; and by 24 h, they had escaped into the cytoplasm (Figure 2F). Biocompatibility assessments at a 2:1 siRNA to dendrimer ratio exhibited no cytotoxicity at 10 µg/mL, while viability remained above 80% at 20 µg/mL (Figure 2G).

The successful synthesis of OD was confirmed by ^1^H nuclear magnetic resonance (^1^H-NMR), with the emergence of the aldehyde peak at 9.63 ppm and a complex set of multiplets between 5.8-4.0 ppm (Figure S8). Based on prior optimization, the hydrogel with 5% (w/v) concentration and a 1:1 G4:OD mass ratio exhibited superior properties and was selected for all subsequent experiments (Figure S9). Injectability was rigorously validated through *in vitro* extrusion assays using 30 G needles, demonstrating maintained structural integrity without phase separation or fragmentation post-extrusion (Figure 2H). Scanning electron microscopy (SEM) imaging revealed a uniformly porous hydrogel microstructure (Figure 2I). Self-healing capability was assessed through a cutting-recovery experiment, where the hydrogel rapidly reformed after cutting attributed to the dynamic reversibility of the Schiff base bonds (Figure 2J). Rapid gelation occurred within minutes upon mixing G4CAsi-FDX1 NPs and OD, as evidenced by shape retention in inverted vials, whereas the hydrogel disintegrated after the addition of 0.1 mM H_2_O_2_ (Figure 2K). The ROS-responsive degradation was assessed by incubating hydrogels in PBS with varying pH (7.4 vs. 6.5) and oxidative conditions (with or without 1 mM H_2_O_2_). The acidic environment marginally accelerated the initial degradation rate through Schiff base hydrolysis, yet ROS presence was still a strong driver of bulk hydrogel disintegration, as comparable ultimate degradation was achieved under neutral oxidative conditions (Figure 2L, S10). The release of CA and si-FDX1 was concomitantly accelerated under acidic and oxidative conditions, validating the synchronized dual-drug release capability of our system in response to pathological stimuli (Figure 2M, S11). Collectively, these results demonstrate the NP-engineered hydrogel as an injectable, self-healing, rapid gelation, and ROS-degradable system.

Beyond these properties, we comprehensively evaluated the hydrogel's mechanical performance to assess its compatibility with the dynamic cardiac tissue environment. Rotational rheometry confirmed the formation of an elastic hydrogel network, characterized by G′ consistently exceeding G″ with clear frequency dependence across 0.1-10 rad/s. Notably, these rheological properties were well maintained after loading both CA and si-FDX1. Strain sweep tests identified the critical strain for network collapse, while step-strain measurements demonstrated rapid self-healing, with over 80% recovery of G′ within 5 seconds after structural disruption (Figure S12). The hydrogel's low Young's modulus (< 1.5 kPa), compared to that of native myocardial tissue (8-16 kPa) 21, 22, serves to minimize compressive damage during injection, avoid restraining contraction, and provide a soft biomimetic microenvironment (Figure S13). Furthermore, ex vivo adhesion tests under simulated physiological conditions demonstrated a shear strength of 3.3 kPa, sufficient to resist detachment caused by cardiac cyclic strain (Figure S14). Coupled with a low swelling ratio (3.11%) and high porosity (~ 85.33%), which mitigate volumetric stress and facilitate metabolite exchange, these mechanical properties collectively affirm the hydrogel's suitability for application in a beating heart.

### Antioxidant and anti-inflammatory effects of G4CAsi-FDX1 NPs

As shown earlier, MIRI induces FDX1 upregulation, which promotes DLAT oligomerization, thereby enhancing catalytic efficiency by increasing enzyme concentration and active sites 23. However, pathological DLAT oligomerization disrupts redox homeostasis, exacerbating ROS production. Specifically, hyperactivation of the α-KGDH complex (converting α-KG to succinyl-CoA) 24 and PDH (converting PA to acetyl-CoA) 25 (Figure S15) elevates NADH/NAD^+^ ratios, leading to electron leakage from the mitochondrial electron transport chain. These surplus electrons react with oxygen to generate superoxide anions (O^2-^), amplifying ROS levels. Concurrently, depletion of iron-sulfur proteins, including aconitase 2 (ACO2) 26 and lipoic acid synthase (LIAS) 27 (Figure S16), diverts metabolic resources toward lipoylation, impairing oxidative phosphorylation and energy homeostasis. Critically, G4CAsi-FDX1 NPs treatment reversed these cascades.

**Figure 3:**
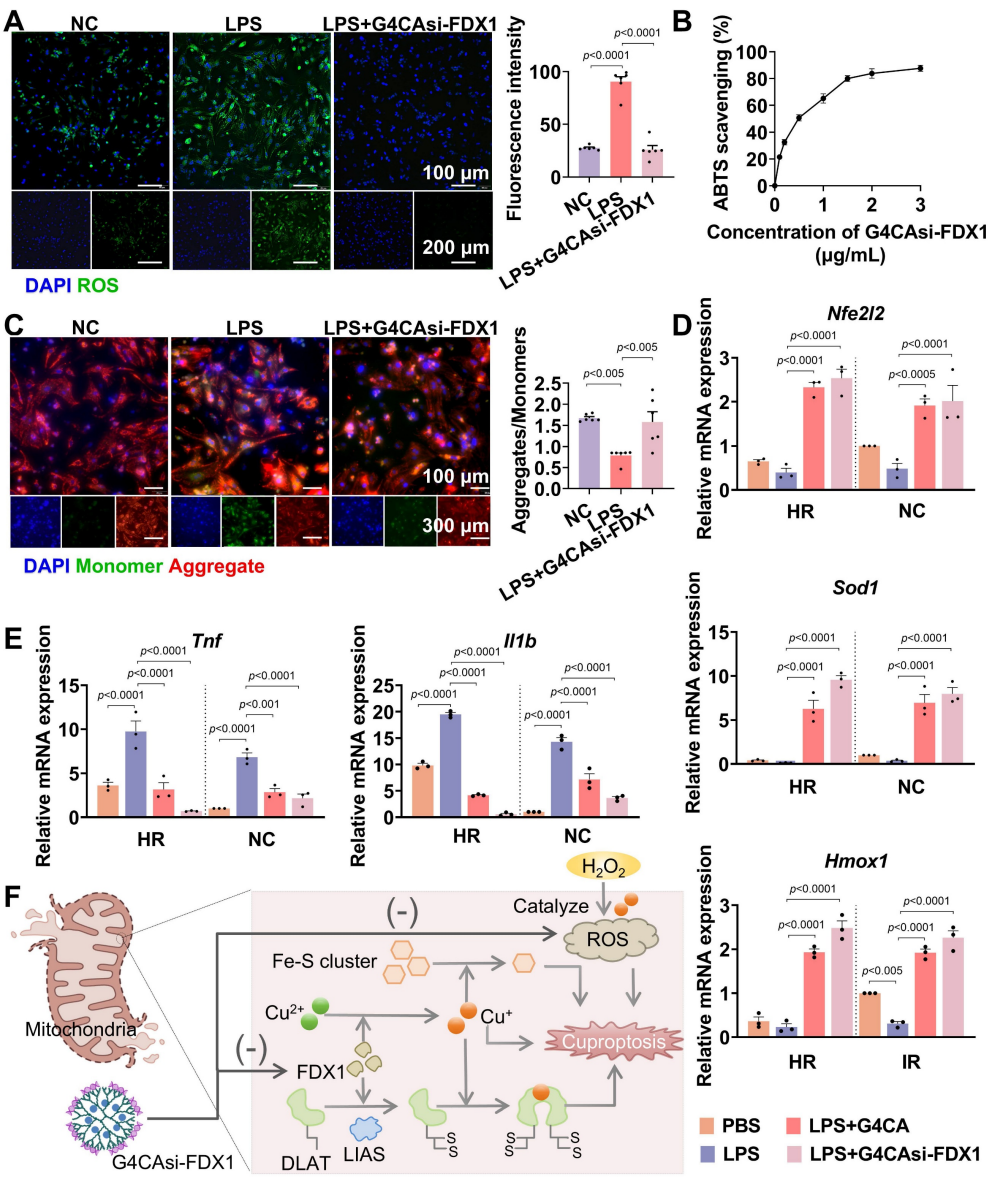
Antioxidant and anti-inflammatory effects of G4CAsi-FDX1 NPs. A) Intracellular ROS levels in NRCMs assessed by ROS probe (green) staining (scale bar: 100 μm and 200 μm) and ROS level quantification in NRCMs with different treatments (n = 6). B) ABTS radical scavenging activity of the various concentration of G4CAsi-FDX1 NPs (n = 3). C) JC-10-based mitochondrial membrane potential assay (scale bar: 100 μm and 300 μm). Red and green fluorescence indicate J-aggregates (polarized) and monomers (depolarized), respectively. Red/green ratio quantification (right, n = 6). qRT-PCR analysis of D) *Nfe2l2* (NRF2), *Sod1* (SOD1), and *Hmox1* (HO1) mRNA levels, and E) *Tnf* (TNF-α) and *Il1b* (IL1β) mRNA levels in NRCMs under the indicated treatments as labeled on the figure (n = 3). F) Illustration of cuproptosis mechanism and G4CAsi-FDX1 NPs' targeting action on FDX1 inhibition (via siRNA-mediated silencing) and ROS scavenging (via CA).

To investigate the therapeutic effects of G4CAsi-FDX1 NPs on oxidative stress and inflammation, key features of MIRI, we exposed primary rat cardiomyocytes to lipopolysaccharide (LPS) stimulation. Treatment with the NPs significantly reduced ROS levels, as evidenced by a substantial decrease in ROS accumulation compared to untreated controls (**Figure 3**A). The 2,2'-azino-bis (3-ethylbenzothiazoline-6-sulfonic acid) (ABTS) radical scavenging assay further confirmed the NPs' antioxidant capacity, showing a notable reduction in free radical levels indicative of effective antioxidant activity (Figure 3B, S17). Additionally, mitochondrial membrane potential assessment using the JC-10 dye revealed that NPs treatment preserved mitochondrial integrity by preventing depolarization, a key indicator of mitochondrial dysfunction (Figure 3C). The mRNA levels of antioxidant factors *Nfe2l2* (nuclear factor erythroid 2-related factor 2, NRF2), *Sod1* (superoxide dismutase 1, SOD1), and *Hmox1* (heme oxygenase 1, HO1) showed a significant increase following NPs treatment (Figure 3D). Conversely, the mRNA levels of pro-inflammatory cytokines *Tnf* (tumor necrosis factor alpha, TNF-α) and *Il1b* (interleukin-1 beta, IL-1β) were reduced, indicating a shift towards an anti-inflammatory state (Figure 3E). Mechanistically, the NPs deliver si-FDX1 to suppress FDX1 expression, thereby reducing Cu^+^ and ROS levels, while CA directly scavenges ROS. Together, these dual mechanisms act synergistically to effectively mitigate oxidative stress (Figure 3F). These results underscore the therapeutic potential of G4CAsi-FDX1 NPs in counteracting the key cellular dysfunctions underlying MIRI.

### Hydrogel's release characteristics and molecular effects in MIRI model

The hydrogel's *in vitro* mechanical properties provide preliminary evidence supporting its potential suitability for dynamic cardiac environments. While our *in vitro* studies have demonstrated initial gel retention and controlled drug release, the long-term stability and functional performance of the hydrogel in complex *in vivo* settings remain to be further validated in future research.

**Figure 4:**
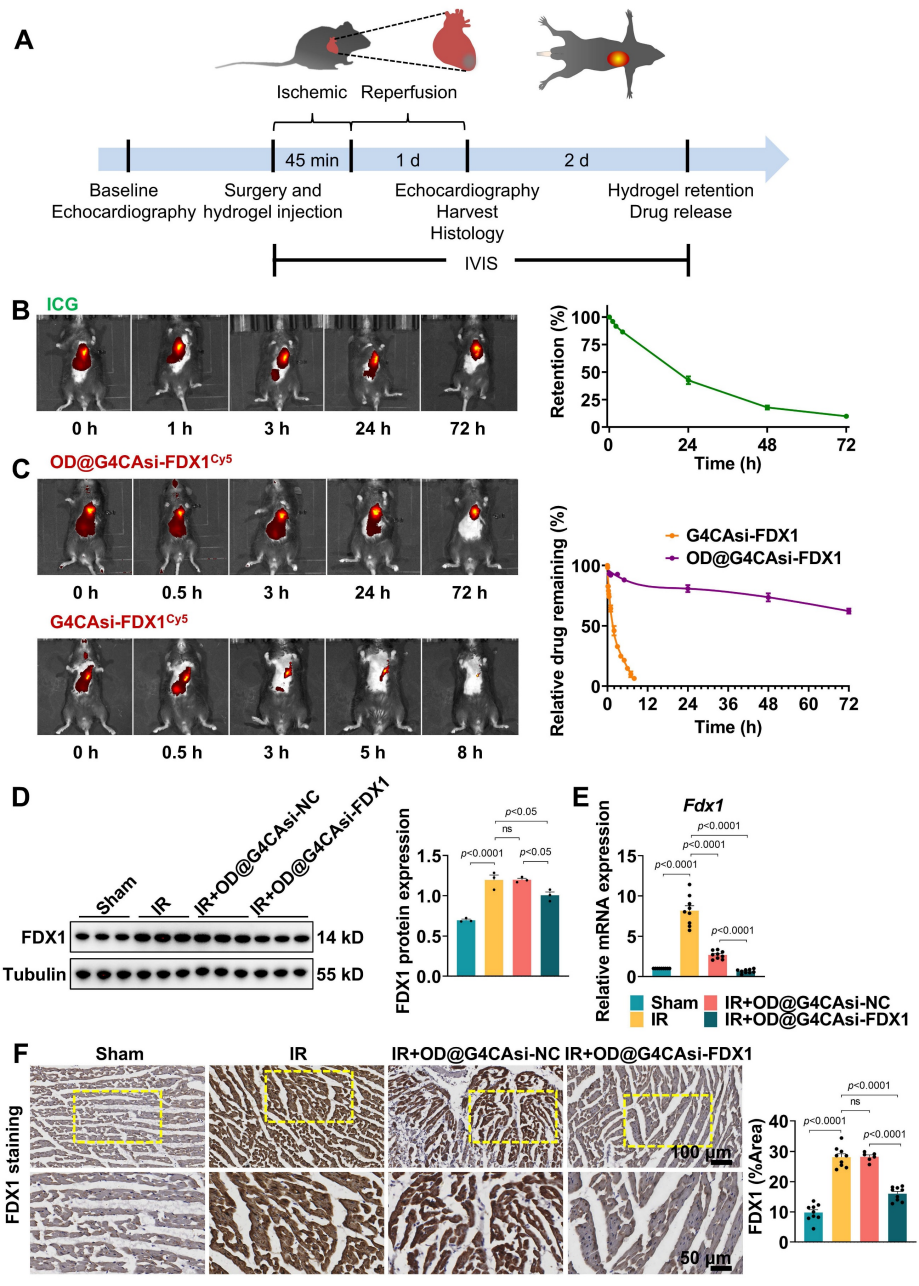
Hydrogel's Release Characteristics and Molecular Effects in MIRI Model. A) Schematic of the mouse I/R model operation flowchart. B) *In vivo* retention of the ICG-labeled hydrogel in mouse myocardium at 0, 1, 3, 24 and 72 h post injection (left). Quantification of hydrogel retention (n = 3, fluorescence intensity normalized to 0 h, right). C) Release kinetics of Cy5-labeled NPs at 0, 0.5, 3, 24 and 72 h post administration (left). Relative drug release profile (n = 3, right). D) Protein levels of FDX1 in heart tissue after different treatments. The left panels are WB bands and the right panels are quantitative data (n = 3). E) qRT-PCR analysis of Fdx1 (FDX1) mRNA level expression (n = 9). F) Immunohistochemistry of FDX1 protein expression in heart sections (scale bar: 100 μm and 50 μm). Quantitative analysis of Average Optical Density (AOD, right, n = 9). AOD was defined as the total optical density divided by the positive staining area. For quantitative data (D-F), data are mean ± s.e.m. Statistical significance is indicated by *p* < 0.05. P values were determined using one-way ANOVA with Tukey's post hoc analysis.

To elucidate whether the hydrogel system can effectively alleviate MIRI, we initially set up a MIRI model using 8-week-old male C57BL/6J mice. MIRI was induced by 45 min occlusion of the LAD followed by 1 day reperfusion (**Figure 4**A). Animals were randomly divided into sham group (n = 6), IR group (n = 6), IR + G4CAsi-NC@OD treated group (n = 6) and IR + G4CAsi-FDX1@OD treated group (n = 6). The respective hydrogels were administered via a single myocardial injection at the onset of reperfusion. All procedures were approved by the Laboratory Animal Ethics Committee of Zhejiang University. IVIS demonstrated the sustained release capabilities of the hydrogel system. Injection of hydrogels labeled with near-infrared dye (ICG) resulted in gradual drug release over 72 h, confirming excellent retention and sustained release at the target site (Figure 4B). Similarly, hydrogels loaded with Cy5-labeled NPs exhibited stable, localized, and controlled drug release over 72 h (Figure 4C). This stability and sustained release profile, enabled by Schiff base chemistry and the encapsulation of functional NPs, offers long-lasting therapeutic effects and potentially reduces the need for frequent administration.

To evaluate the impact of the hydrogel system on key molecular targets, we first assessed its effects on FDX1 expression. Notably, both FDX1 and DLAT oligomer levels were significantly reduced in OD@G4CAsi-FDX1-treated groups compared to the IR group or the OD@G4CAsi-NC group (Figure 4D, S18). Furthermore, FDX1 mRNA expression was markedly suppressed in the OD@G4CAsi-FDX1 group, indicating the effective siRNA-mediated gene silencing (Figure 4E). Notably, DLAT mRNA remained suppressed across all IR groups and was not restored by treatment (Figure S19), indicating that the reduction of toxic DLAT oligomers occurs post-translationally via prevented FDX1-mediated lipoylation, rather than through transcriptional regulation. Immunohistochemical (IHC) analysis further confirmed the significant reduction of FDX1 and DLAT levels in myocardial tissues of the OD@G4CAsi-FDX1 treated group compared to controls (Figure 4F, S20), underscoring its targeted action in FDX1-driven cuproptosis. Functioning as a targeted delivery platform, this hydrogel system translates the NPs' synergistic molecular mechanism into a sustained therapeutic action within the infarct zone, thereby breaking the self-amplifying ROS-copper injury cycle and positioning itself as a promising strategy against MIRI.

### Therapeutic efficacy of ROS-responsive hydrogel in MIRI

**Figure 5:**
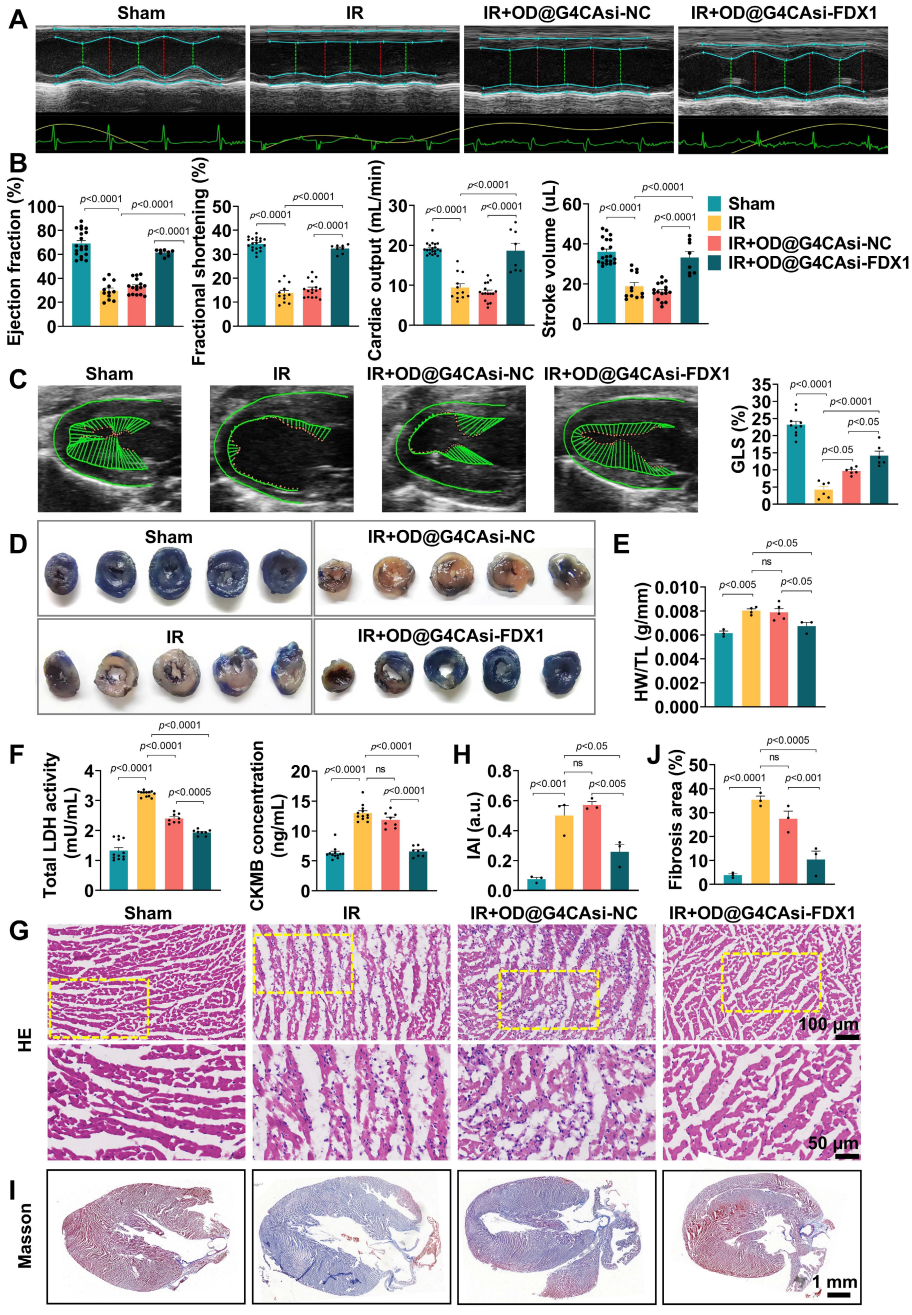
Therapeutic Efficacy of ROS-responsive Hydrogel in a Mouse MIRI Model. A, B) Echocardiography and measured EF%, FS%, CO%, SV% (n = 12). C) The left panels are representative longitudinal 2D B-mode images and strain traces across different groups. The right panels are quantitative result of GLS (n = 6). D) Representative images of heart sections stained with TTC. E) Heart weight to tibia length ratio (HW/TL). F) Serum levels of LDH and CK-MB in mice at day 1 postinjection (n = 8). G, H) Representative images of HE staining of the heart tissues from mice at day 1 postinjection (scale bar: 100 μm and 50 μm) and quantitative analysis of inflammatory cell infiltration (n = 3). I, J) Representative images of Masson's staining (scale bar: 1mm) and measured fibrosis area% (n = 3).

To assess the therapeutic efficacy of our ROS-responsive hydrogel system, we performed comprehensive echocardiographic analysis on mice subjected to MIRI. As shown in **Figure 5**A and 5B, OD@G4CAsi-FDX1 hydrogel treatment resulted in significant improvements in left ventricular ejection fraction (LVEF), fractional shortening (FS), cardiac output (CO), and stroke volume (SV), compared to the IR injury group. These improvements were further confirmed by myocardial deformation analysis, with quantitative assessment of global longitudinal strain (GLS), which visually demonstrated the protective effects of the OD@G4CAsi-FDX1 hydrogel treatment on cardiac contractility (Figure 5C, S21). Histological analysis revealed a marked reduction in infarct size in the OD@G4CAsi-FDX1 group compared to the IR group, as assessed by Evans blue and triphenyl tetrazolium chloride (TTC) staining (Figure 5D). In addition, the heart weight-to-tibia length ratio (HW/TL), a key indicator of cardiac function and remodeling, was significantly improved in the OD@G4CAsi-FDX1-treated group (Figure 5E). Furthermore, serum levels of myocardial injury markers, lactate dehydrogenase (LDH) and creatine kinase-MB (CK-MB), were notably decreased in the OD@G4CAsi-FDX1 group, highlighting the hydrogel's effectiveness in mitigating myocardial damage (Figure 5F). Inflammatory responses were also reduced in OD@G4CAsi-FDX1 treated animals, as evidenced by a decreased Inflammatory Activity Index (IAI) and a reduction in inflammatory cell infiltration observed in hematoxylin and eosin (HE)-stained sections (Figure 5G, H). Further IHC and IF analysis showed a reprogrammed immune microenvironment, characterized by reduced macrophage infiltration and a decreased M1/M2 ratio. This shift from a pro-inflammatory M1 phenotype in the IR group to a pro-reparative M2 phenotype after hydrogel treatment (Figure S22, S23). Masson's trichrome staining revealed that, in the MIRI model, the collagen content in the infarcted area was significantly increased, whereas the fibrotic region was notably reduced after hydrogel injection (Figure 5I, J).

Collectively, these findings demonstrate that the therapeutic efficacy of the OD@G4CAsi-FDX1 hydrogel arises through a coordinated multi-level mechanism. Upon localization to the infarct site via ROS-responsive release, the hydrogel enables a synergistic two-stage action, scavenging acute oxidative stress while concurrently inhibiting FDX1-mediated cuproptosis. This dual intervention preserves cardiomyocyte integrity, thereby reducing infarction, inflammation, and fibrosis, ultimately leading to robust functional recovery. Together, these results underscore the potential of this comprehensive strategy for combatting myocardial injury and enabling repair.

Future studies employing single-cell RNA sequencing will be essential to delineate cell type-specific responses and to further elucidate the molecular mechanisms underlying the therapeutic efficacy of OD@G4CAsi-FDX1 hydrogel. While our study demonstrates the promising therapeutic potential of the OD@G4CAsi-FDX1 hydrogel, several key challenges must be addressed to advance its clinical translation. These include ensuring consistent drug release across heterogeneous patient microenvironments, establishing long-term biosafety profiles, scaling up manufacturing under GMP compliance, and developing minimally invasive catheter-based delivery strategies alongside biomarker-guided patient selection. Future work will focus on these critical aspects, including the development of multi-stimuli responsive formulations, rigorous large-animal testing, and process optimization, which are essential steps toward clinical application.

The promising therapeutic effects observed in murine models establish a robust foundation for exploring translational potential. To advance toward clinical application, future studies should prioritize validating the system in large animal models (e.g., pigs or canines), which exhibit greater anatomical and physiological similarity to human cardiac structures, particularly in coronary vasculature, myocardial thickness, and hemodynamic profiles. These models more accurately simulate the clinical context of MI/R therapy and facilitate the exploration of more clinically relevant delivery routes.

Furthermore, exploring integration with percutaneous coronary intervention (PCI), the standard of care for MI/R, could enhance translational relevance. Speculatively, the ROS-responsive system could be administered pre-PCI as a protective pretreatment. During the critical window after coronary guidewire crossing but before balloon inflation or stent deployment, hydrogel injection may precondition the myocardium to attenuate impending reperfusion injury, leveraging transient coronary access without delaying revascularization. Alternatively, post-PCI delivery could sustain cytoprotection during the 24-72-h peak injury phase, utilizing the hydrogel's sustained release profile. Combined with catheter-guided targeted delivery, this strategy could synergize with revascularization efforts to address both acute injury and long-term remodeling, potentially mitigating infarct expansion and reducing hospital stays.

## Conclusions

This study establishes a multifunctional ROS-responsive hydrogel platform designed to synergistically combat MIRI by concurrently targeting cuproptosis and oxidative stress. Engineered via dynamic Schiff base bonds between OD and G4, the hydrogel achieves spatiotemporally controlled drug release triggered by pathological ROS levels. Co-loaded with si-FDX1 and CA, it executes dual therapeutic actions: si-FDX1 silences FDX1 to block Cu^2+^-to-Cu^+^ conversion and subsequent lipoylated protein aggregation, while CA directly scavenges ROS, synergistically mitigating oxidative damage and inflammation. *In vivo* studies validated the hydrogel's efficacy. Following a single myocardial injection, the treatment significantly reduced infarct size and improved cardiac function, alongside sustained drug release and demonstrating high biocompatibility. By synchronizing drug release with ROS fluctuations, this platform overcomes the limitations of conventional single-mode therapies, addressing both upstream cuproptosis and downstream oxidative stress. In summary, our findings suggest that the ROS-responsive hydrogel represents a compelling therapeutic strategy for treating MIRI. Given its ROS-responsiveness and cuproptosis-targeting capacity, the hydrogel paves the way for future investigations into medicine approaches for ischemic heart diseases.

## Supplementary Material

Supplementary materials and methods, figures.

## Figures and Tables

**Scheme 1:**
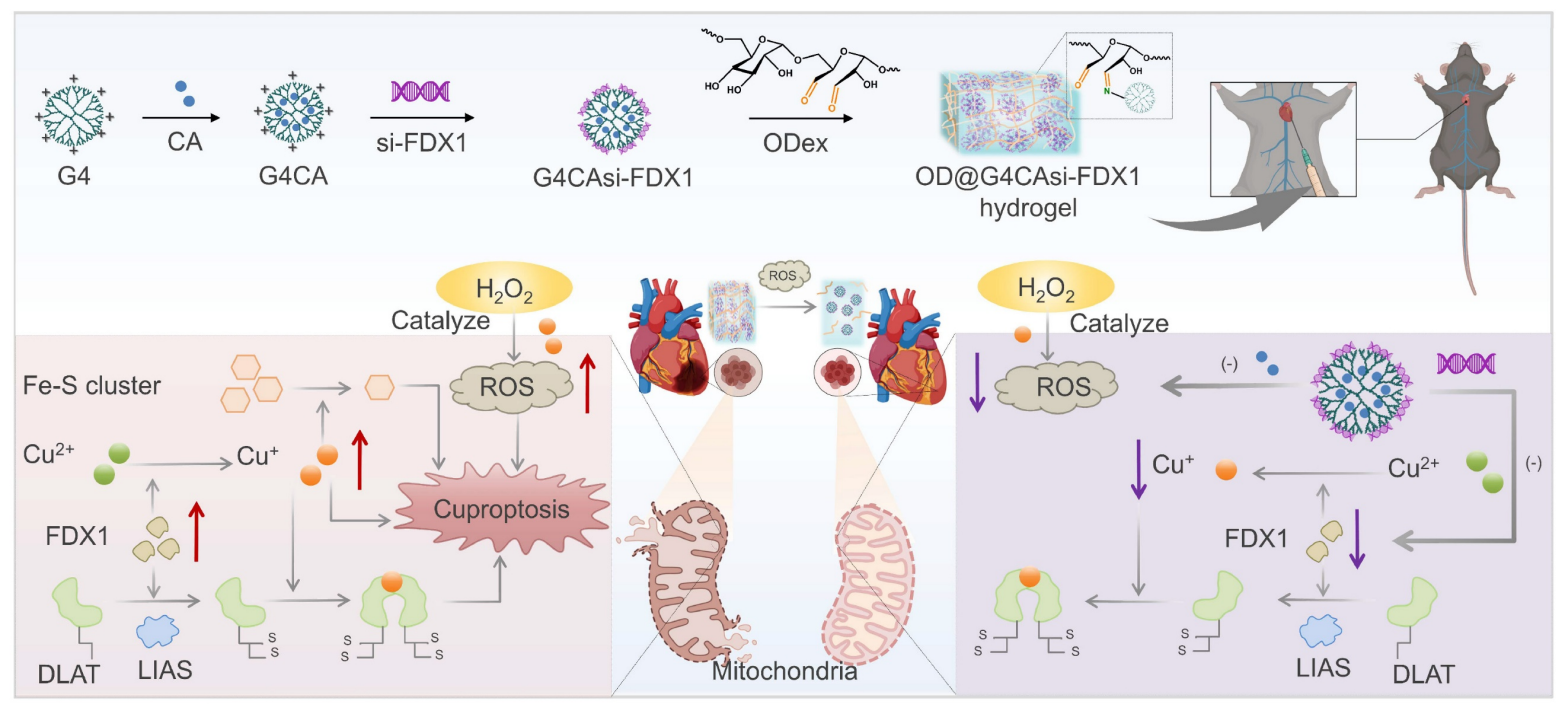
Injectable hydrogel OD@G4CAsi-FDX1 releases CA (ROS scavenger) and si-FDX1 (cuproptosis inhibitor) upon ROS-triggered degradation, synergistically repairing MIRI.

## Abbreviations

MIRI: myocardial ischemia-reperfusion injury
MI: myocardial infarction
ROS: reactive oxygen species
TCA: tricarboxylic acid
FDX1: Ferredoxin 1
CA: caffeic acid
PAMAM G4: fourth-generation polyamidoamine
si-FDX1: FDX1-targeted siRNA
OD: oxidized dextran
GEO: Gene Expression Omnibus
LAD: left anterior descending coronary artery
DEGs: differentially expressed genes
GSEA: Gene set enrichment analysis
GO: Gene Ontology
KEGG: Kyoto Encyclopedia of Genes and Genomes
GO-BP: biological process
GOCC: cellular component
GO-MF: molecular function
EE: Encapsulation efficiency
NRCMs: Neonatal rat cardiomyocytes
DMEM: Dulbecco's Modified Eagle Medium
FBS: fetal bovine serum
WB: Western blot
qRT-PCR: Real-time fluorescence quantitative polymerase chain reaction
CCK-8: Cell counting kit-8
ICG: Indocyanine Green
IVIS: in vivo imaging system
HR: hypoxia-reoxygenation
DLAT: dihydrolipoamide S-acetyltransferase
ɑ-KGDH: alpha-ketoglutarate dehydrogenase
PDH: pyruvate dehydrogenase
ɑ-KG: alpha-ketoglutarate
PA: pyruvate
cTnT: cardiac troponin T
NPs: nanoparticles
DLS: dynamic light scattering
TEM: transmission electron microscopy
^1^H-NMR: ^1^H nuclear magnetic resonance
SEM: scanning electron microscopy
ACO2: aconitase 2
LIAS: lipoic acid synthase
LPS: lipopolysaccharide
ABTS: 2,2'-Azino-bis (3-ethylbenzothiazoline-6-sulfonic acid)
NRF2: nuclear factor erythroid 2-related factor 2
SOD1: superoxide dismutase 1
HO1: heme oxygenase 1
TNF-ɑ: tumor necrosis factor alpha
IL-1β: interleukin-1 beta
IHC: immunohistochemistry
AOD: average optical density
LVEF: left ventricular ejection fraction
FS: fractional shortening
CO: cardiac output
SV: stroke volume
TTC: triphenyl tetrazolium chloride
HW/TL: heart weight-to-tibia length ratio
LDH: lactate dehydrogenase
CK-MB: creatine kinase-MB
IAI: inflammatory activity index
HE: hematoxylin and eosin
PCI: percutaneous coronary intervention
GLS: global longitudinal strain
