# Supplementary material for: Precision targeting of FDX1-mediated cuproptosis by a ROS-responsive hydrogel for myocardial ischemia-reperfusion injury treatment
Source: Theranostics. 2026 Jan 1;16(3):1281–94. doi: 10.7150/thno.120455 (PMC12679365; doi:10.7150/thno.120455)
Supplement: Supplementary file 1 — Supplementary materials and methods, figures. [file thnov16p1281s1.pdf]

Precision Targeting of FDX1-Mediated Cuproptosis by a ROS-Responsive Hydrogel for Myocardial Ischemia-Reperfusion Injury Treatment

Jiayi Hu[1], Xiaoyi Bao[1], Meihua Ting[1], Yecheng Tao[1], Ran Li[1], Guosheng Fu[1], Fuyu Qiu[1*], Jing Zhao[1*], Wenbin Zhang[1*]

1 Zhejiang Key Laboratory of Cardiovascular Intervention and Precision Medicine, Engineering Research Center for Cardiovascular Innovative Devices of Zhejiang Province, Department of Cardiology, Sir Run Run Shaw Hospital, Zhejiang University School of Medicine, Hangzhou, 310016, China.

*Emails: 3309029@zju.edu.cn (F. Qiu), jingzhao123@zju.edu.cn (J. Zhao), 3313011@zju.edu.cn (W. Zhang)

Supporting Information

Methods

Data acquisition pre-processing

Microarray gene expression profiles of heart ventricles from GSE193997 were obtained from Gene Expression Omnibus (GEO) (https://www.ncbi.nlm.nih.gov/geo/) based on the GPL17021 platform. Rats underwent surgery for LAD ligation for 45 min followed by reperfusion, and the heart ventricle tissues of rats were collected at 6H after reperfusion. Firstly, we used the Geoquery function in R language (V4.3.0) to download the dataset. The gene probe was transformed into gene symbols according to the annotation information for GPL17021 and normalized for differentiation analysis.

Identification of differentially expressed genes (DEGs)

DEGs between MIRI and sham groups were identified using the limma R package, which is an efficient analysis method in bioinformatics. The selected criteria were set as the values of $|logFC| > 0.5$ and P-value < 0.05. The packages of ggplot2 and pheatmap in R were employed to plot the heatmaps and volcano plot of DEGs, respectively.

Gene set enrichment analysis (GSEA)

The normalized expression data for all genes were acquired to conduct GSEA. GSEA was performed to identify the pathways that were significantly enriched between the MIRI and control groups according to the "clusterProfiler" package. If a gene set had a positive enrichment score, then the set was termed "enriched" which implied that the majority of these genes in the set had higher expression and were accompanied by a higher risk score.

Gene Ontology (GO) and Kyoto Encyclopedia of Genes and Genomes (KEGG) functional enrichment analyses

All DEGs screened under conditions of P-value < 0.05, were subjected to GO analysis for allocation to relevant GO terms, including GO-BP (biological process), GOCC (cellular component), and GO-MF (molecular function). Particularly, gene symbols of DEGs were converted to gene IDs according to the "org.Mm.eg.db" package; the latter were subsequently used for functional enrichment analysis according to the "clusterProfiler" package. Details of the genes belonging to particular GO terms with their fold change values are presented as circus plots using the "Goplot" package. As previous GO analysis, gene symbols of DEGs were converted to gene IDs according to the "org.Mm.eg.db" package; the latter were subsequently used for KEGG pathway analysis according to the"clusterProfiler" package.

Oxidation degree of oxidized dextran (OD)

The oxidation degree of OD was determined using a titration method based on the reaction between aldehyde groups (-CHO) and hydroxylamine hydrochloride ($NH_2OH \cdot HCl$), which generates oxime (R-CH=NOH) and releases equimolar H^+ ions. The released H^+ was titrated with standard NaOH to calculate the oxidation degree. Briefly, 0.2 g of OD sample was added to 50 mL of 0.25 M hydroxylamine hydrochloride solution containing 0.01% methyl orange, and the mixture was incubated at room temperature for 15-30 min. The solution was then titrated with 0.1 M NaOH until the indicator changed from red to yellow (pH ≈ 4.4), with a blank titration (without OD) performed simultaneously to correct for background. The oxidation degree was calculated using the formula: Oxidation degree (%) = $(\Delta V \times C_{NaOH} \times M_w / m_0) \times 100\%$, where ΔV represents the volume difference of NaOH consumed between sample and blank titration (L), C_{NaOH} is the concentration of NaOH (0.1 mol/L), m_0 is the mass of OD sample (0.2 g), and M_w is the molecular weight of the dextran repeating unit (g/mol).

Optimization of Hydrogel Formulation

To optimize the OD@G4CAsi-FDX1 hydrogel, gelation performance was first assessed using the test tube tilting method. Hydrogel samples with varying G4:OD mass percentages (5%, 4%, 3%, 2.5%) were prepared. The 5% G4:OD hydrogel maintained its shape, while 4% was slightly loose, and 2.5% and 3% failed to gel, flowing down the test tube. Thus, 5% was selected for further rheological and mechanical testing.

Hydrogels with G4:OD mass ratios of 3:7, 4:6, 5:5, 6:4, and 7:3 were prepared, and subjected to frequency sweep rheology and compressive modulus tests. The 5:5 ratio exhibited the highest storage modulus (G') and Young's modulus, indicating superior gel network stability, mechanical strength, and elasticity. Therefore, the 5:5 G4:OD ratio was selected as optimal for mechanical performance.

Swelling Ratio Measurement

The swelling ratio of the hydrogel was determined gravimetrically. Freshly prepared hydrogels (initial weight, M_0) were immersed in deionized water and incubated at 37-50 °C for 24 h to reach equilibrium swelling. The swollen hydrogels were then removed, blotted gently with filter paper to remove excess surface water, and immediately weighed (M_s). The swelling ratio was calculated using the following equation: Swelling Ratio (%) = $(M_s - M_0) / M_0 \times 100\%$

Porosity Measurement

The porosity of the hydrogels was measured using a compression-weight method. The weight of the hydrogel in the equilibrium swollen state was recorded as W_{sw}. The swollen hydrogel was then thoroughly compressed between sheets of filter paper three times to remove water from the macropores, and the weight was immediately measured as W_{sq}. Assuming the density of the swollen hydrogel (ρ_{sw}) approximates that of water (ρ_{H_2O}), the porosity (P) was calculated as: P (%) = $(1 - W_{sq} / W_{sw}) \times 100\%$.

Rheological Characterization

The viscoelastic properties of the hydrogels were analyzed using a rotational rheometer. Oscillatory frequency sweep tests were performed from 0.1 to 10 rad/s at a fixed strain amplitude of 5.0% to determine the storage (G') and loss (G") moduli. Strain amplitude sweep tests (γ = 0.1% to 1000%) were conducted to identify the critical strain point. Alternating step strain tests were carried out at 37 °C and a constant frequency of 1 Hz, switching between a small strain (γ = 5.0%) for 60 s and a large strain (γ = 1000%) for 60 s, to evaluate the self-healing capability. All measurements were performed on three formulations: OD@G4, OD@G4CA, and OD@G4CAsi-FDX1, with 3-5 replicates per group.

Mechanical Compression Testing

The compressive mechanical properties were evaluated using a universal testing machine equipped with a 500 N load cell. Cylindrical hydrogel samples (10 mm in diameter, 10 mm thick) were compressed at a constant rate of 10 mm/min. The compressive modulus was determined from the linear elastic region of the resulting stress-strain curve.

Ex Vivo Adhesion Assessment

Cardiac tissues were pre-hydrated in phosphate-buffered saline (PBS) or normal saline and stored in sealed bags at 4 °C prior to testing. For shear adhesion testing, a hydrogel sample (10 × 10 mm) was sandwiched between two strips of cardiac tissue (30 × 10 mm) and pressed under a pressure of ~1.5 kPa for 30 s to form an adhesive bond. The assembled construct was then equilibrated in a humid environment for 24 h to allow the hydrogel to reach swelling equilibrium before testing. The shear adhesion strength was then measured using a universal testing machine.

Protein expression analysis via Western blot (WB)

After cell digestion and centrifugation, RIPA lysis buffer containing 1 mM PMSF was added to the cell pellet, followed by lysis on ice for 30 min. The lysate was then centrifuged at 12,000 rpm for 15 min to

collect the supernatant. Protein concentrations were determined using a BCA protein assay kit, and the proteins were denatured by adding SDS-PAGE loading buffer and heating at high temperature. Equal amounts of denatured proteins were loaded onto a gel for electrophoresis at 80V for 2 h using a Bio-Rad Protein Electrophoresis and Blotting System. Proteins were subsequently transferred to a PVDF membrane at 300 mA for 1.5 h. The membranes were blocked with TBST buffer containing 5% skimmed milk (0.5% Tween-20 in TBS buffer) for 2 h at room temperature. Membranes were then incubated with primary antibodies overnight at 4°C, followed by incubation with species-specific secondary antibodies for 1 h at room temperature. During the incubation steps, the membranes were washed three times with TBST for 5 min each. After final incubation with ECL working solution, protein bands were detected using the Bio-Rad ChemiDoc MP Imaging System and analyzed with Image J software. For non-reducing SDS-PAGE, RIPA lysis buffer was replaced with IP lysis buffer, and SDS-PAGE loading buffer was replaced with non-reducing loading buffer (devoid of reducing agents such as β-mercaptoethanol or DTT). All other steps remained consistent with the standard protocol described above.

Real-time fluorescence quantitative polymerase chain reaction (qRT-PCR)

Total mRNA was isolated from the tissues or cells using MolPure® TRIeasy™ Plus Total RNA Kit (YEASEN, Shanghai, China), and cDNA was prepared using RT (YEASEN, Shanghai, China) according to the manufacturer's instructions. The mRNA levels were assessed on a QuantStudio™ 6 Flex System (ABI, USA) by qRT-PCR using Hieff® qPCR SYBR® Green Master Mix (YEASEN, Shanghai, China). The results were analyzed and calculated by $2^{-\Delta\Delta Ct}$ method. Gene expression was normalized against β-actin.

Detection of cell viability

Cell counting kit-8 (CCK-8) assay (C0039, Beyotime Biotechnology) was utilized for test NRCM cell viability. Briefly, cells were cultivated into 96-well plate (10000 cells per well) and exposed to si-FDX1 or PBS under normoxia or hypoxia condition for 2 h, and then transferred to normal incubator for 24 h. Then, 10 μL kit solution was mixed into the culture medium for 1 h. The absorbance was tested by Microplate reader (PerkinElmer EnSpire, MA, USA) at 450 nm. Cell viability was expressed as the percentages of control.

HE staining

Tissue samples were dehydrated in a sucrose solution at 4°C until the tissue settled. The dehydrated tissue was then embedded in OCT compound and frozen. Frozen sections of 5 μm thickness were cut using a cryostat. The sections were rehydrated, followed by staining with hematoxylin for 5 min. After washing with water, the sections were stained with eosin for 2 min. Finally, the sections were dehydrated through graded ethanol, cleared with xylene, and mounted with a coverslip for observation under a microscope.

Masson's Trichrome staining

Tissue sections (5 μm) were prepared from paraffin-embedded samples. After deparaffinization and rehydration through a series of graded alcohols, sections were stained with Masson's Trichrome staining kit. Briefly, the sections were stained with hematoxylin for 5 min, followed by a differentiation step in 1% hydrochloric acid for 1 min. After washing, the sections were stained with ponceau acid fuchsin for 10 min, followed by phosphomolybdic acid for 10 min. The sections were then stained with aniline blue solution for 5 min. After dehydration in graded alcohols and clearing in xylene, the sections were mounted with a coverslip and observed under a light microscope.

Immunohistochemistry

Tissues prepared either from paraffin-embedded samples (5 μm thickness) were stained by immunohistochemistry with various primary antibodies including a non-immune IgG, followed by further staining with secondary antibodies.

Statistical analysis

SPSS20.0 statistical software was used. Data were shown as mean determinants ± s.e.m.. Mann-Whitney test was used to analyze variables with skewed distribution while the unpaired Student's t-test was used to assess differences between two groups for data with normal distributions. For comparison of multiple groups, ANOVA was utilized. Counting data are expressed as frequency and percentage and chi-square test is used. Measurement data were expressed as median (quartile), and Mann Whitney U test was used for comparison between groups. There was statistical difference of 0.05. Kaplan-Meier survival curves delineate differences in major cardiovascular adverse events between the two groups.

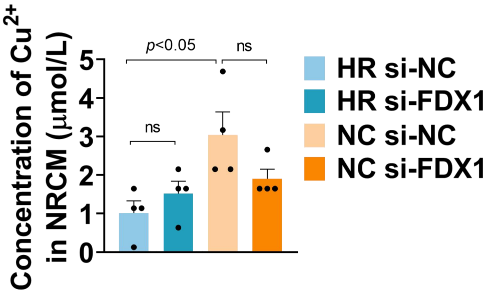

Figure S1. Relative concentration of Cu^{2+} in NRCMs.

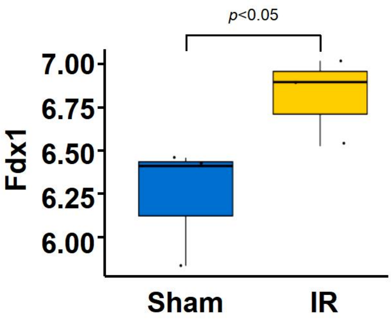

Figure S2. Expression levels of FDX1 in the sham operation group and the IRI group were illustrated by box plot. Statistical significance is indicated by $p < 0.05$.

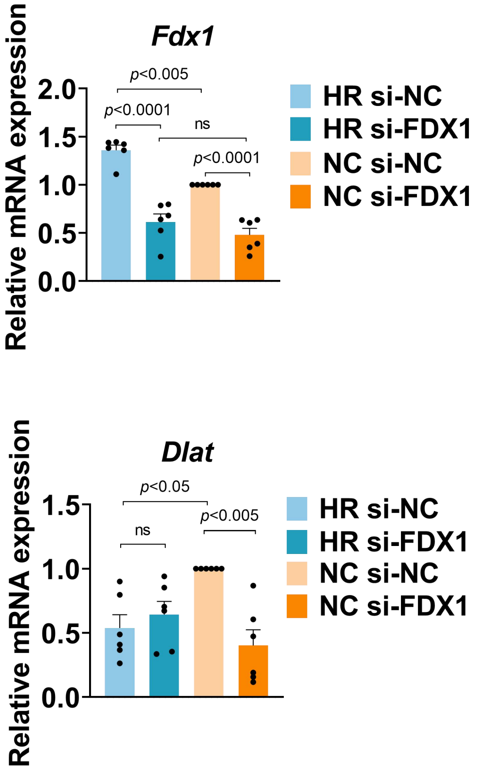

Figure S3. Relative mRNA levels of *Fdx1* (FDX1) and *Dlat* (DLAT) in NRCMs were checked by qRT-PCR.

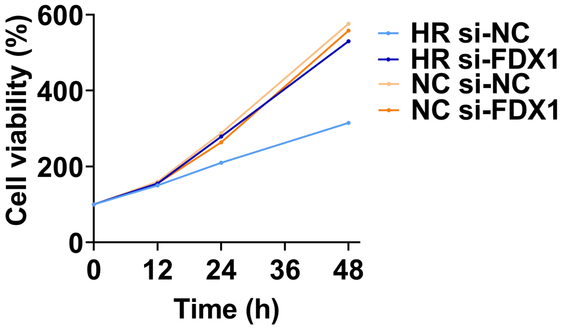

Figure S4. Cell viability was measured with the CCK8 assay.

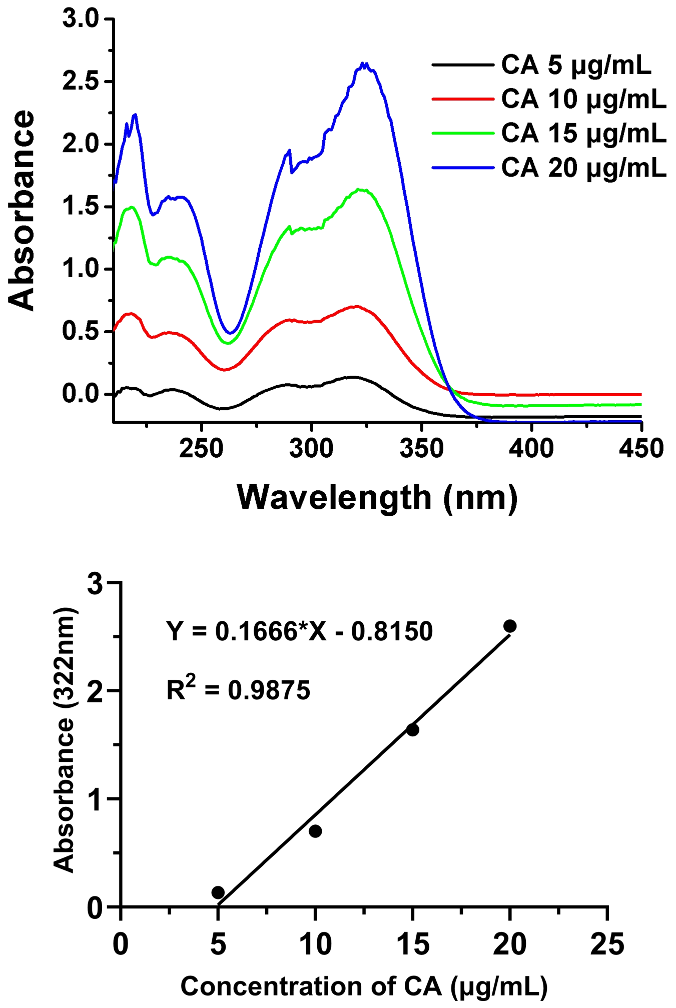

Figure S5. The UV-vis spectra and standard curve of CA.

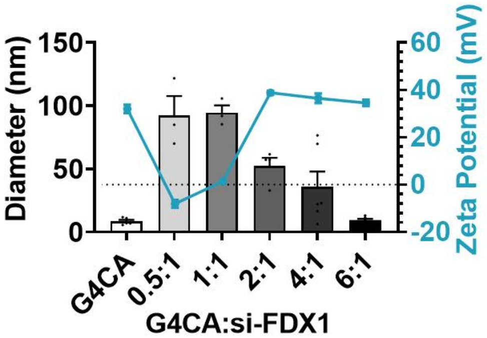

Figure S6. The particle sizes of G4CA and G4CAsi-FDX1 with different ratios were measured by DLS.

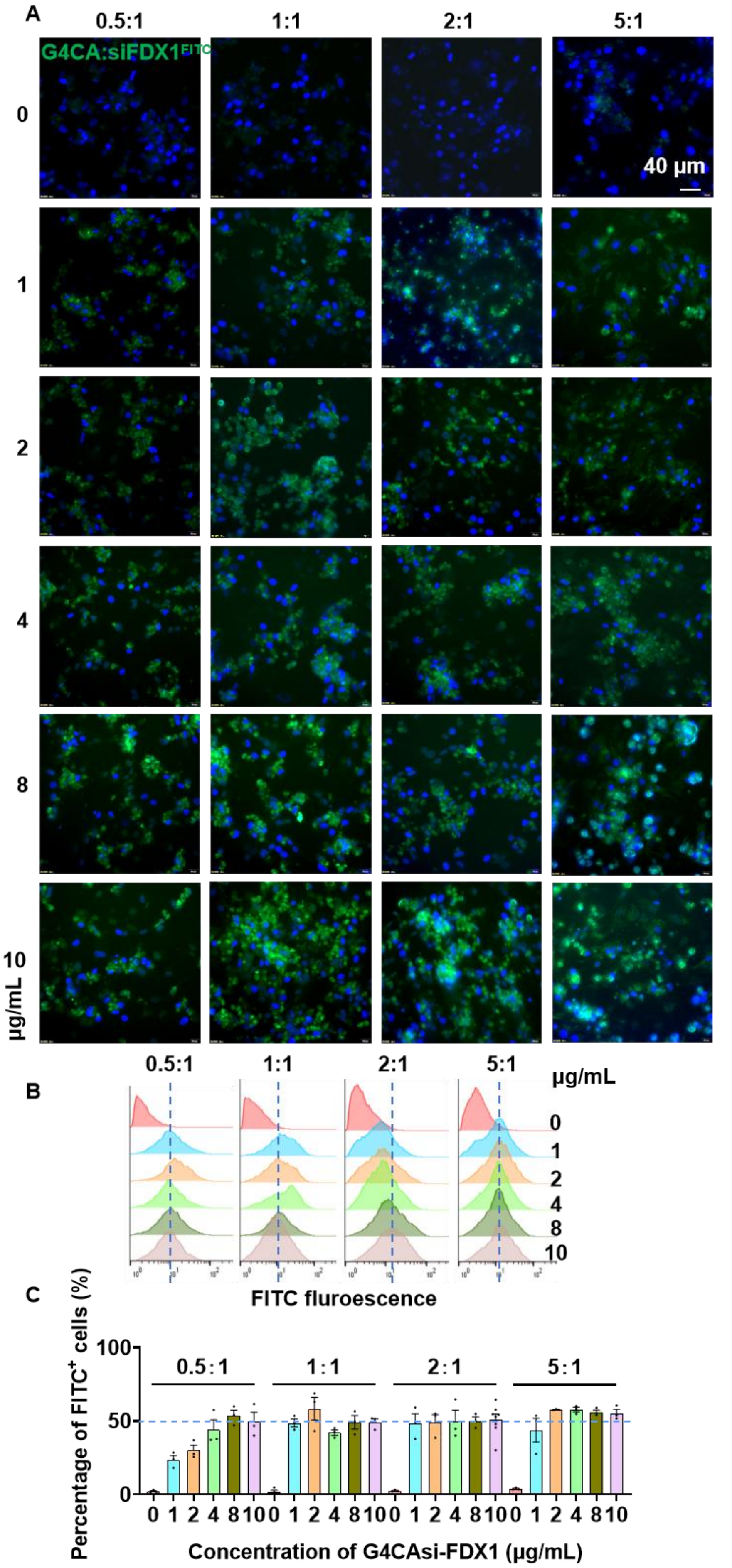

Figure S7. Cellular Uptake of G4CAsi-FDX1 NPs. A) Representative images of cellular uptake of G4CAsi-FDX1 NPs at different concentrations and with varying ratios of G4CA to FITC-labeled si-FDX1 in NRCMs (scale bar: 40 μm). B) Flow cytometric profile and C) quantitative results.

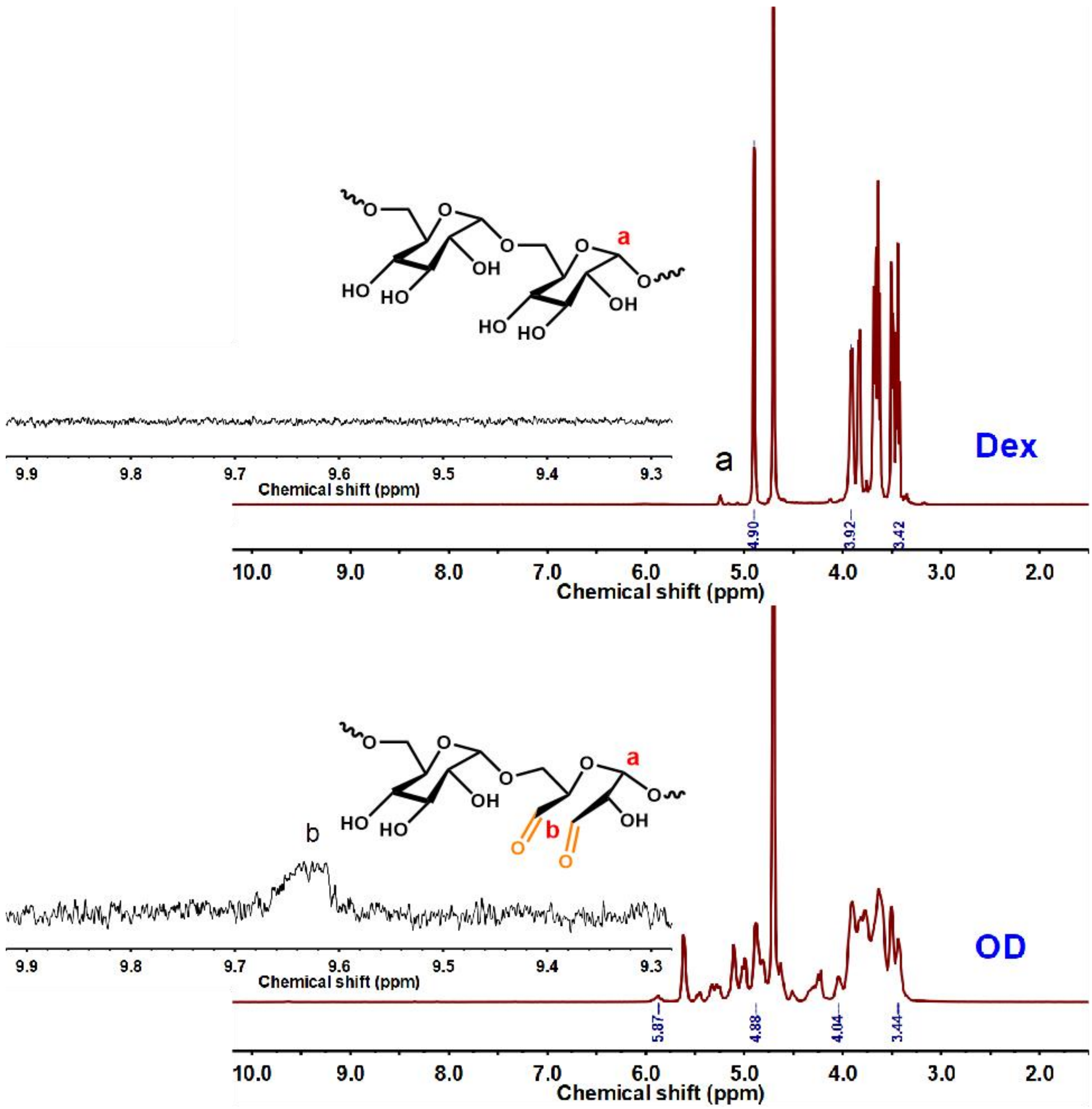

Figure S8. ^{1}H NMR of Dex and OD.

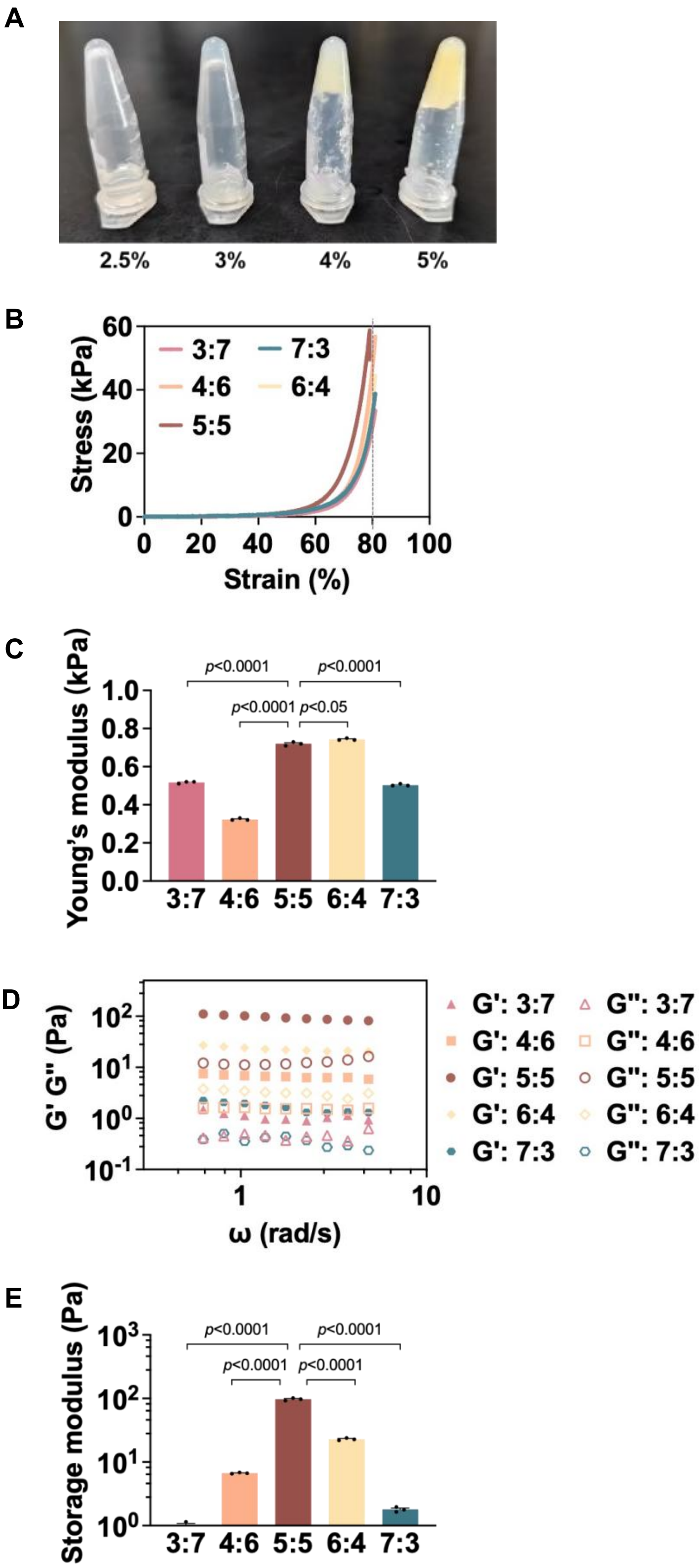

Figure S9. Optimization of Hydrogel Formulation. A) Images of 2.5-5% (w/v) hydrogels formed at a 1:1 G4:OD mass percentages. B) Compressive stress-strain curves of fully swollen hydrogels. C) Young's modulus derived from the linear region (0-20% strain) of the curves (n = 3). D) Frequency sweep of the hydrogel at 0.1-10 rad/s and 1.0% strain. E) Storage modulus comparison at 1 rad/s (n = 3).

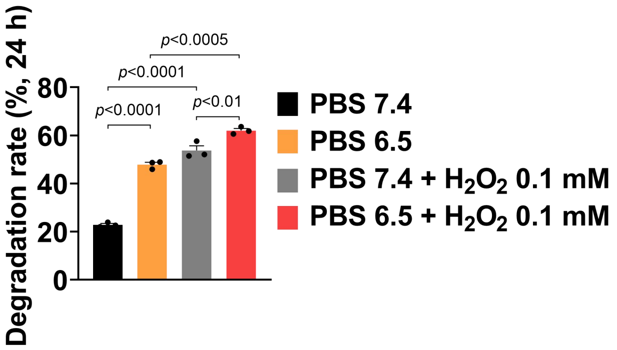

Figure S10. Comparative hydrogel degradation after 24 h under different pH and oxidative conditions.

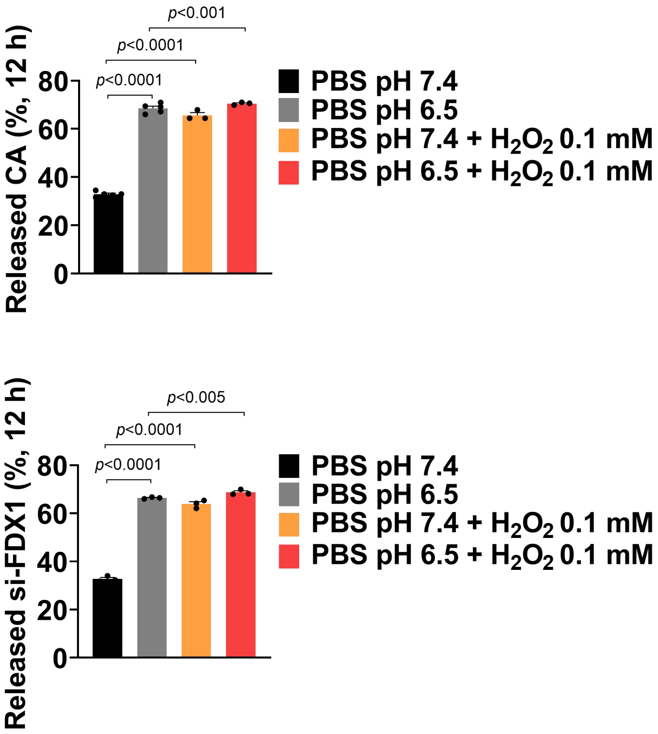

Figure S11. Comparative CA and si-FDX1 release after 12 h under different pH and oxidative conditions.

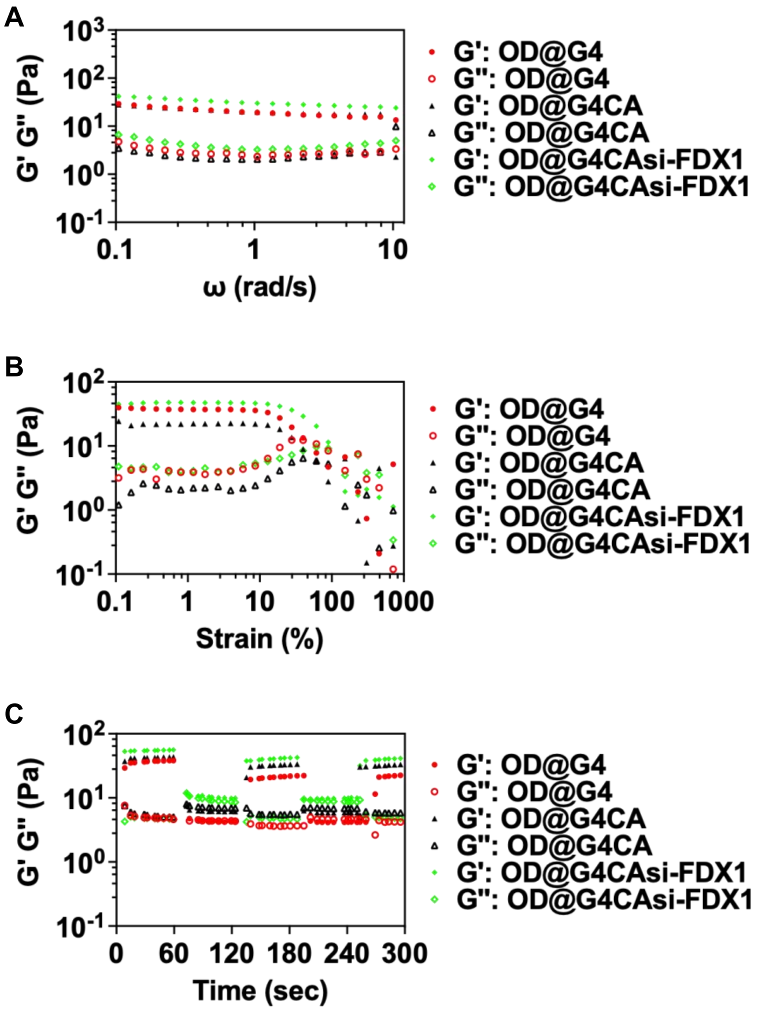

Figure S12. Rheological Characterization of Hydrogels. A) Frequency sweep of the hydrogel at 0.1-10 rad/s and 5.0% strain. B) Strain amplitude sweep of the hydrogel (γ = 0.1-1000%) at 1 Hz. C) Step-strain measurements of the hydrogel alternating between 5% and 1000% strain at 1 Hz.

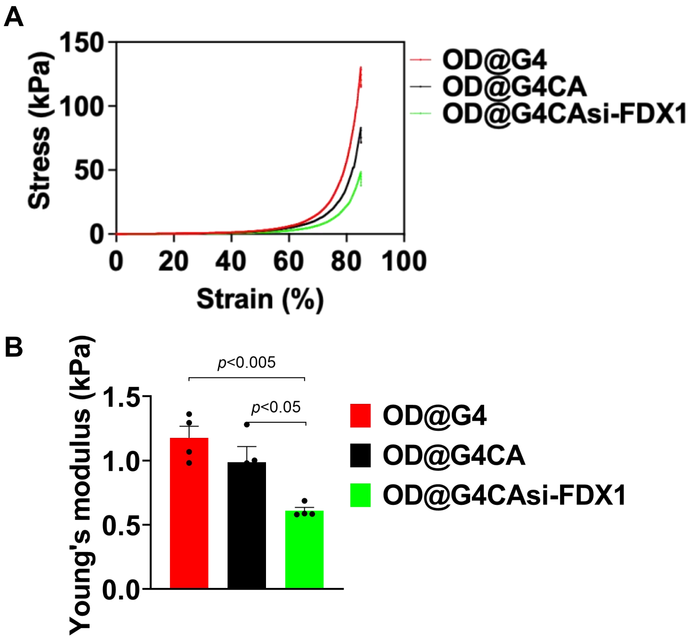

Figure S13. Mechanical Compression Testing. A) Compressive stress-strain curves of fully swollen hydrogels. B) Young's modulus derived from the linear region (5-15% strain) of the curves (n = 4).

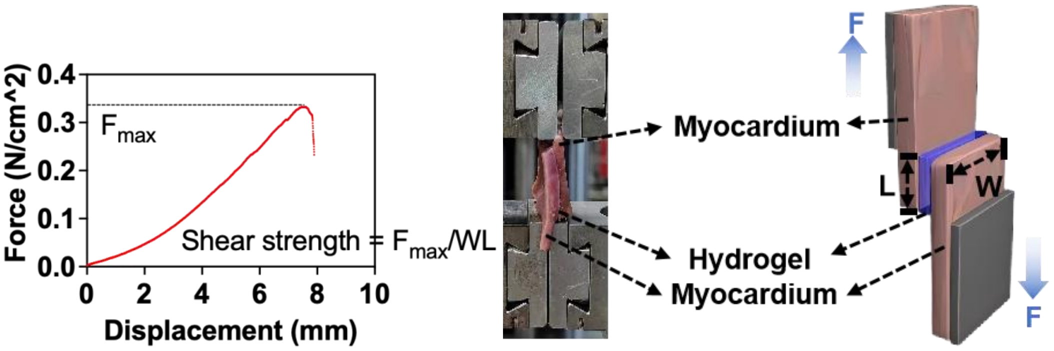

Figure S14. Shear adhesion strength of the hydrogel on cardiac tissue.

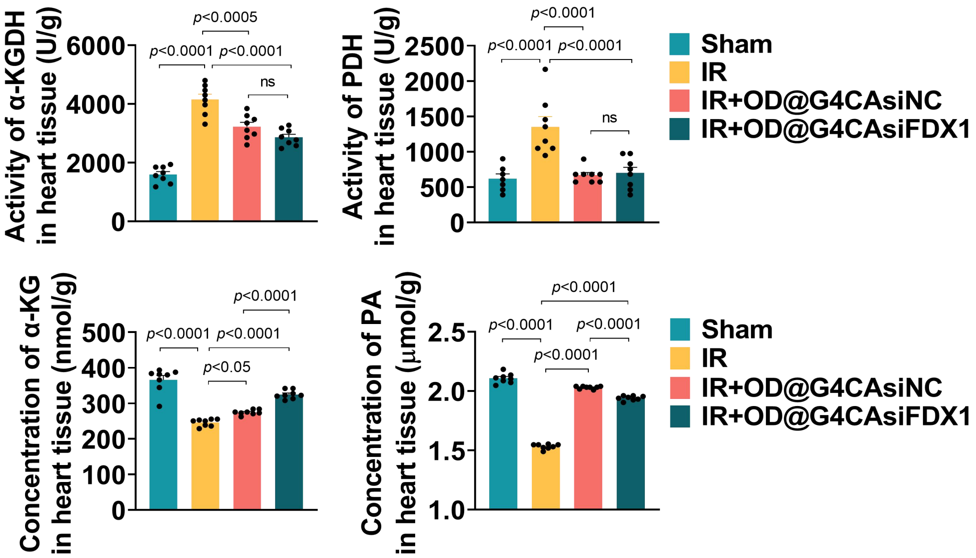

Figure S15. Activity of α-KGDH and PDH in heart tissues after different treatments, and concentration of α-KG and PA in heart tissues after different treatments.

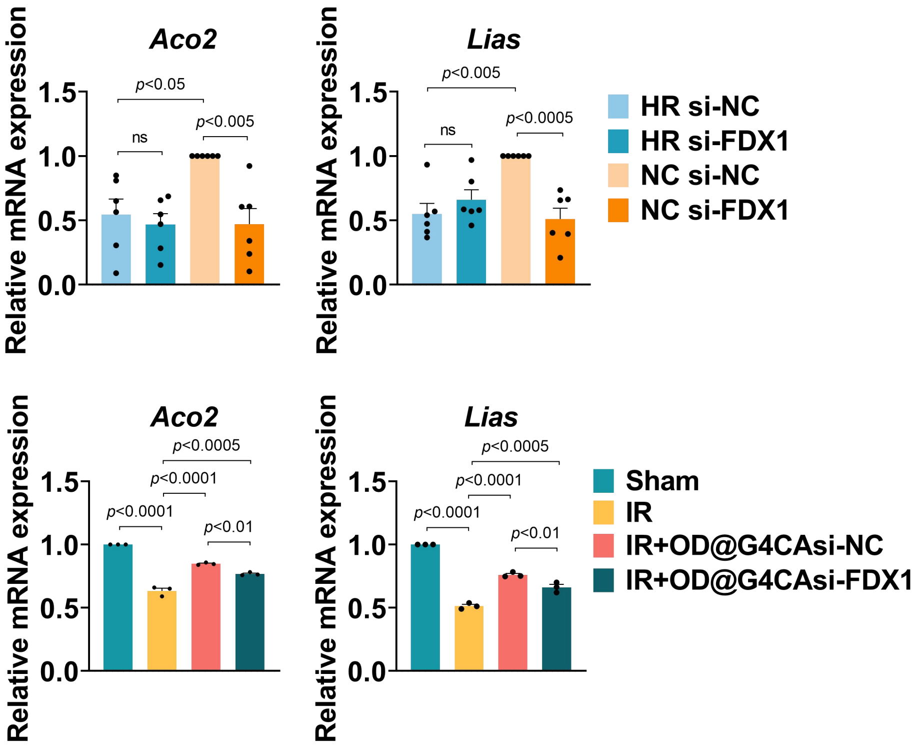

Figure S16. qRT-PCR analysis of *Aco2* (ACO2) and *Lias* (LIAS) mRNA levels in NRCMs and in heart tissue under different treatments.

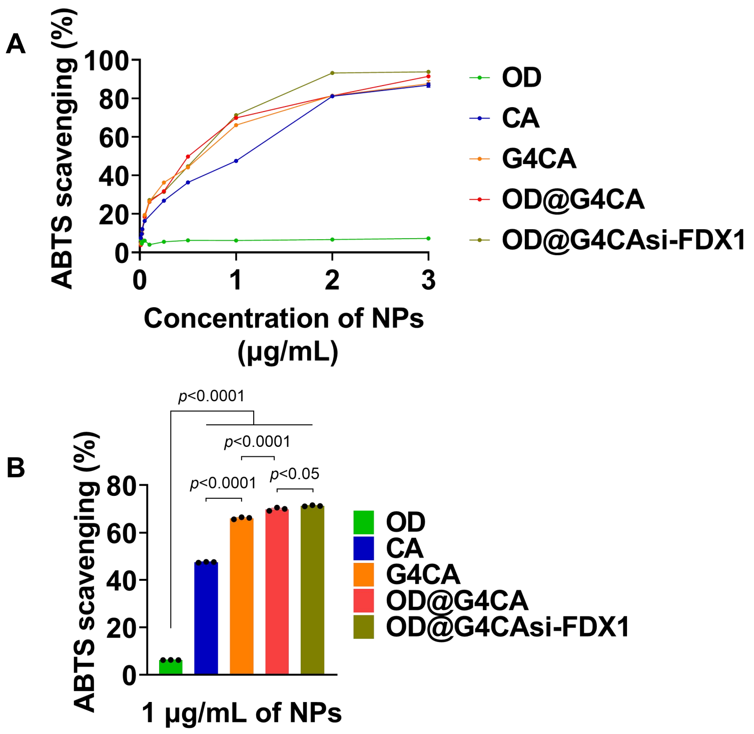

Figure S17. ABTS radical scavenging activity of the NPs. A) ABTS radical scavenging activity of the various concentration of OD, CA, G4CA, OD@G4CA, OD@G4CAsi-FDX1 and B) the scavenging rates of all formulations at 1 µg/mL.

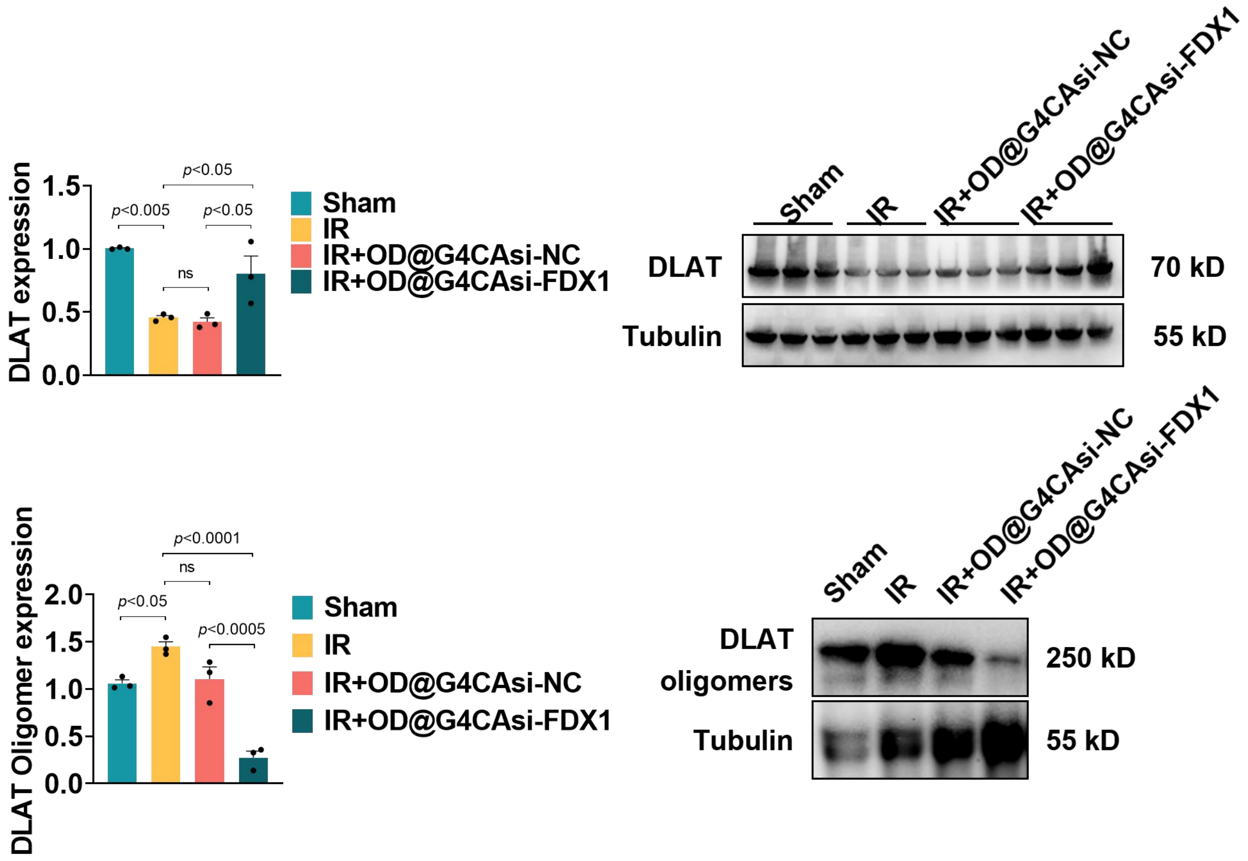

Figure S18. Protein levels of DLAT (70 kD) and DLAT oligomers (250 kD) in heart tissue after different treatments. The left panels are quantification results and the right panels are representative Western Blot bands.

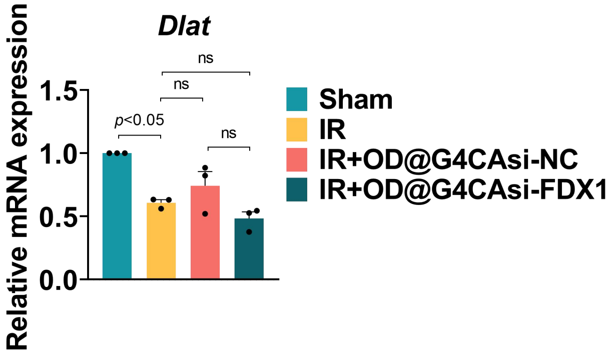

Figure S19. qRT-PCR analysis of *Dlat* (DLAT) mRNA levels in NRCMs and in heart tissue under different treatments.

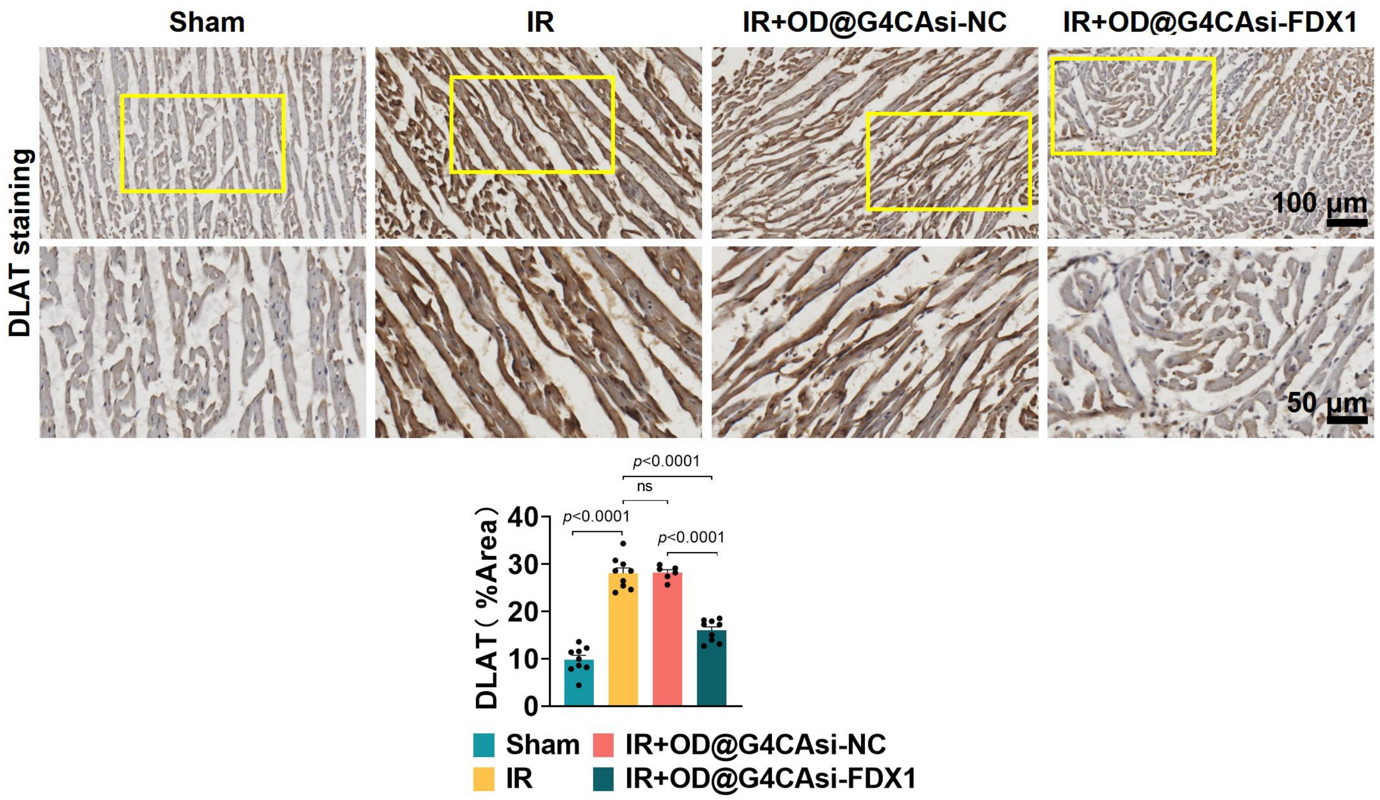

Figure S20. Immunohistochemistry of heart sections (scale bar: 100 μm and 50 μm). Quantitative analysis of AOD (n = 3).

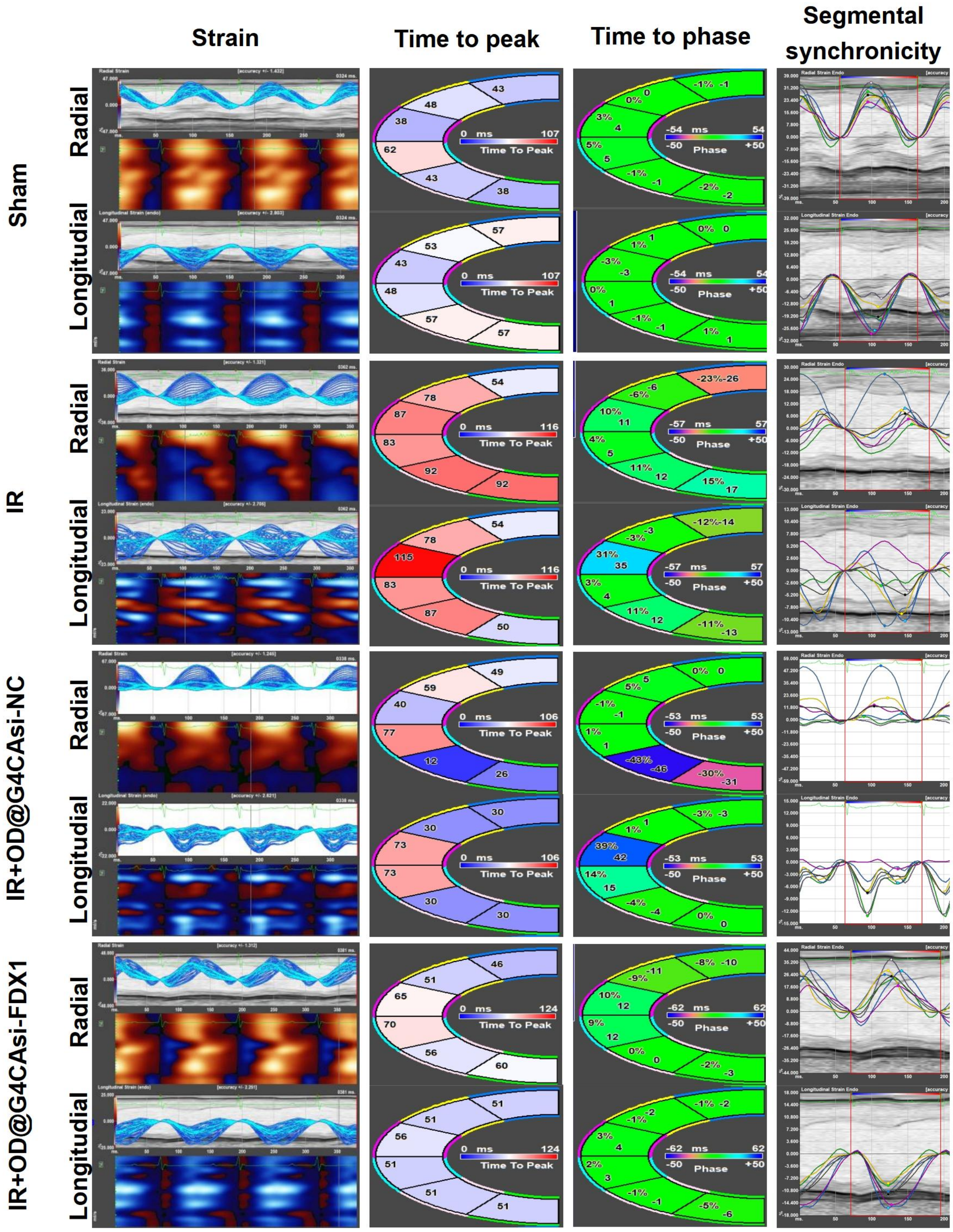

Figure S21. Speckle tracking-based echocardiography representatives of mice at different treatments (from left: 2-dimensional [2D] strain, time to peak, time to phase, segmental synchronicity graph with displayed curves).

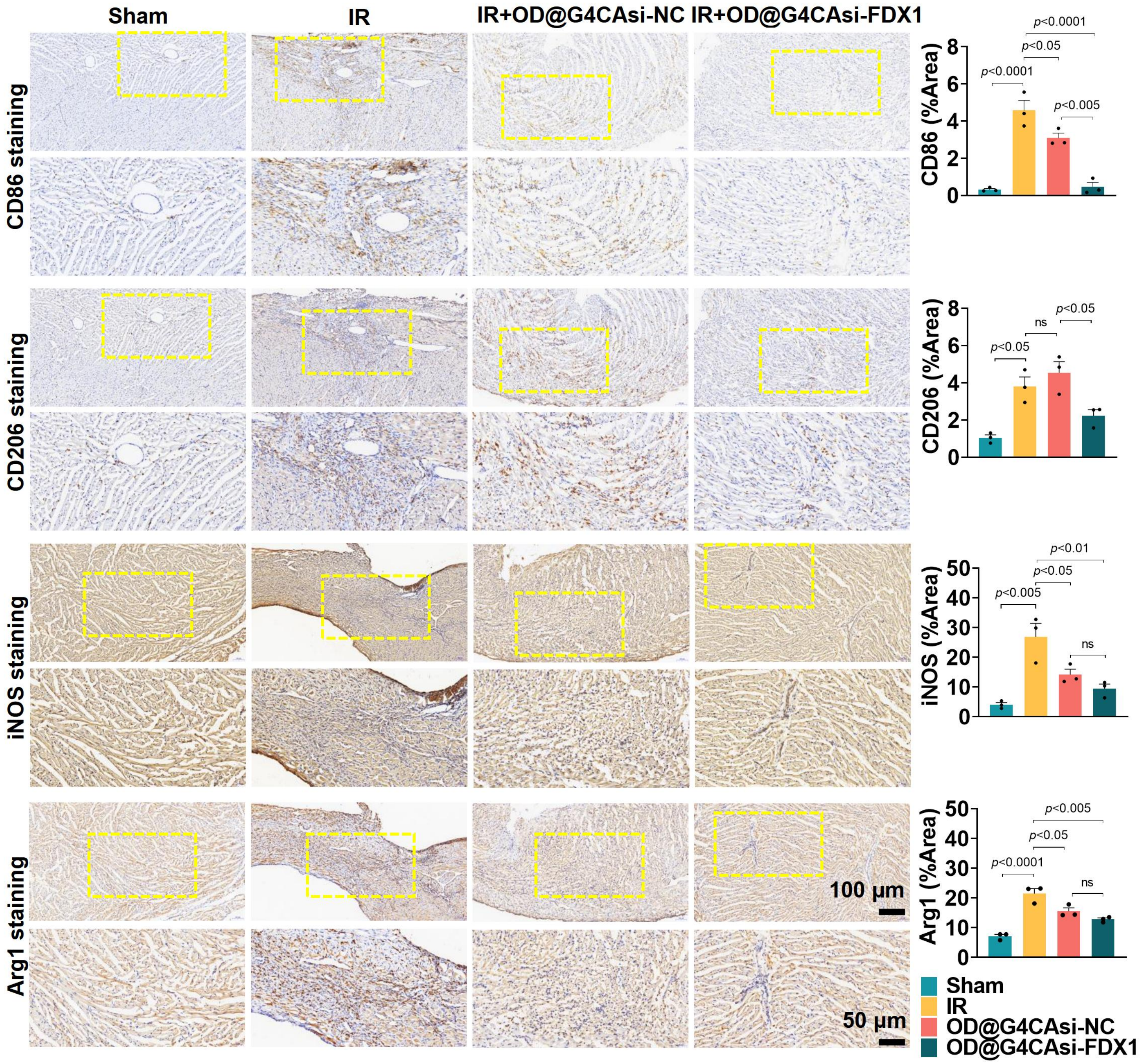

Figure S22. Representative immunohistochemistry images of CD86, iNOS, CD206, and Arg1 in myocardial sections (scale bars: 100 μm and 50 μm). Quantitative analysis of Average Optical Density (AOD) for each marker (AOD = total optical density/positive area).

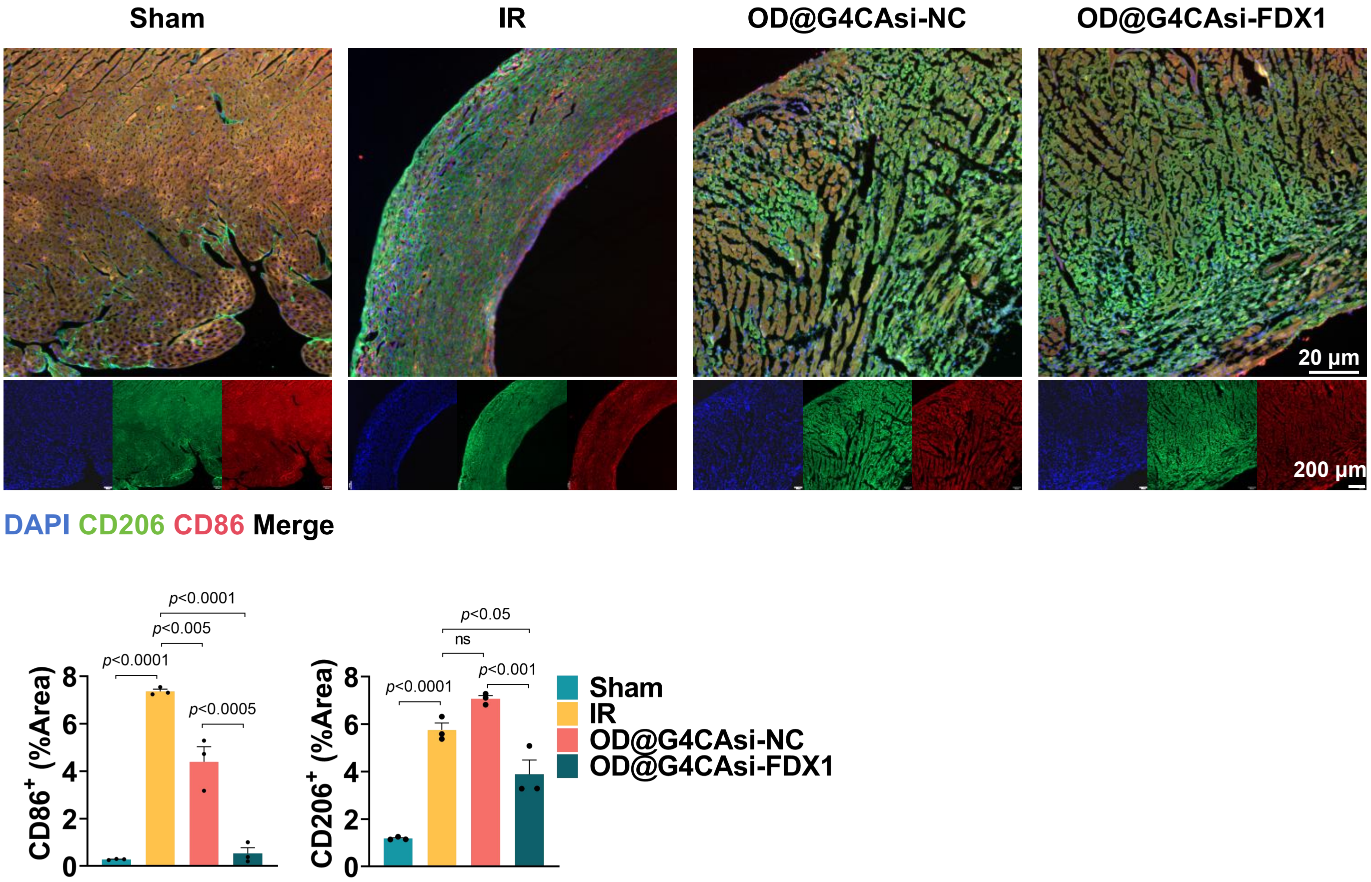

Figure S23. Representative immunofluorescence images showing $CD86^+$ (red, M1 macrophage) and $CD206^+$ (green, M2 macrophage) cells in the infarct border zone. Nuclei are counterstained with DAPI (blue). Scale bar: 200 μm. Quantitative analysis of the percentage area positive for CD86 and CD206 (n = 3).
